# Effects of diets containing proteins from fish muscles or fish by-products on the circulating cholesterol concentration in rodents: a systematic review and meta-analysis

**DOI:** 10.1017/S000711452200349X

**Published:** 2023-08-14

**Authors:** Maria O’Keeffe, Oddrun Anita Gudbrandsen

**Affiliations:** Dietary Protein Research Group, Centre for Nutrition, Department of Clinical Medicine, University of Bergen, 5021 Bergen, Norway

**Keywords:** Rat, Mouse, Fish fillet, Fish residuals

## Abstract

A high circulating cholesterol concentration is considered an important risk factor for the development of CVD. Since lean fish intake and fish protein supplementation have been associated with lower cholesterol concentration in some but not all clinical studies, the main aim of this study was to investigate the effect of diets containing proteins from fish muscles and fish by-products on the serum/plasma total cholesterol (TC) concentration in rodents. A systematic literature search was performed using the databases PubMed, Web of Science and Embase, structured around the population (rodents), intervention (type of fish and fraction, protein dose and duration), comparator (casein) and the primary outcome (circulating TC). Articles were assessed for risk of bias using the SYRCLE’s tool. A meta-analysis was conducted in Review Manager v. 5·4·1 (the Cochrane Collaboration) to determine the effectiveness of proteins from fish on the circulating TC concentration. Thirty-nine articles were included in the systematic review and meta-analysis, with data from 935 rodents. The risk of bias is unclear since few of the entries in the SYRCLE’s tool were addressed. Consumption of proteins from fish resulted in a significantly lower circulating TC concentration when compared with control groups (mean difference −0·24 mmol/l, 95 % CI − 0·34, −0·15, *P* < 0·00001), with high statistical heterogeneity (I^2^ = 71 %). To conclude, proteins from fish muscles and by-products show promise as a functional dietary ingredient or supplement by preventing high cholesterol concentration in rodents, thus reducing one of the most important risk factors for developing CVD.

CVD is one of the leading causes of death worldwide^(1)^. An elevated circulating cholesterol concentration is one of the major modifiable risk factors associated with CVD^(2,3)^, and is also linked with long-term risk of CHD, and all-cause mortality^(4,5)^. Circulating cholesterol is a strong predictor of vascular mortality, especially for ischaemic heart disease^(6)^, and lowering serum cholesterol concentration is an important strategy for reducing the risk of CVD. The primary prevention strategy for CVD includes lifestyle modifications and the use of lipid-lowering drugs to target risk factors^(7)^. Due to poor compliance over time and the patients’ unwillingness to tolerate even mild side effects of statins^(8)^, non-pharmacological treatment of hypercholesterolaemia is the recommended first approach^(7,9)^. Dietary intervention should be the desirable tactic as it is cost effective and includes multiple benefits without the potential side effects of common drugs.

Observational studies consistently show that a high fish consumption is associated with a lower risk of CVD^(10,11)^, and eating fish at least once per week significantly reduces the risk of cardiovascular mortality^(12)^. The benefits of fish consumption on CVD risk are often accredited to the intake of long-chain *n*-3 PUFAs^(13)^; however, these fatty acids do not affect cholesterol concentration in humans^(14–16)^ and lower the cholesterol concentration in rats and mice only when given in very high doses^(17)^. Interestingly, favourable outcomes, including higher HDL-cholesterol^(18–21)^ and lower LDL-cholesterol^(22)^ concentrations, have been demonstrated in some clinical studies investigating the effects of lean fish intake or supplementation with cod protein powder. The reported effects of lean fish intake on cholesterol concentration in humans are inconsistent, and a recent systematic review and meta-analysis called out the need for studies of better quality to clarify the impact of lean fish and fish proteins on the lipid profile^(23)^.

The circulating cholesterol concentration is influenced by several factors, including cholesterol intake, endogenous cholesterol synthesis, cholesterol uptake by the liver and extrahepatic tissues, as well as the excretion of cholesterol and bile acids in faeces. In humans, the cholesterol homeostasis is usually described based on serum/plasma concentrations of total cholesterol (TC), LDL-cholesterol and HDL-cholesterol, whereas in experimental animals we also have the opportunity to measure the daily excretion of cholesterol and bile acids in faeces under controlled conditions and to quantify the cholesterol content and the amounts or activities of relevant enzymes in the liver. Many pre-clinical studies have investigated the effects of fish or fish protein consumption on cholesterol metabolism. Often, but not consistently, the literature differentiates between lean and fatty fish species, but as of yet, there are no reviews or meta-analyses summarising the effects of specific fish species or fish fractions, i.e. muscles (fillet) *v*. by-products, which could potentially explain the inconsistencies observed. The main aim of this systematic review and meta-analysis was to investigate the effect of intake of diets containing proteins from fish muscles and fish by-products on serum/plasma TC concentration in rodents. The secondary aim was to provide a narrative synthesis of the findings from the included articles structured around the population (rodents), the intervention (type of fish and fraction, protein dose and duration), the comparator (casein as control protein) and composition of diets (regular rodent diet, high-fat diet, high-carbohydrate diet, diet added cholesterol alone or in combination with cholate) on the circulating concentrations of TC, HDL-cholesterol and LDL-cholesterol, the hepatic content of TC, the daily faecal excretion of TC and total bile acids, and on four essential proteins involved in the cholesterol metabolism in the liver; i.e. cholesterol 7 alpha-hydroxylase (CYP7A1) which is the rate-determining enzyme for the conversion of cholesterol to bile acids, HMG-CoA reductase which is the rate-determining enzyme for the hepatic synthesis of cholesterol, the LDL-receptor which binds to the apolipoproteins apoB100 and apoE, and sterol O-acyltransferase 2 (SOAT2, also known as acyl-CoA:cholesterol acyltransferase) which catalyse the esterification of cholesterol. A better understanding of these factors is important for the design of future studies in humans and animals targeting the effects of fish and fish proteins on cholesterol metabolism.

## Methods

### Protocol and registration

The review protocol can be viewed at the International prospective register of systematic reviews (PROSPERO) website (https://www.crd.york.ac.UK/PROSPERO), with registration number CRD42022308735.

### Search strategy

A comprehensive literature search was carried out in accordance with the Preferred Reporting Items for systematic reviews and Meta-Analyses (PRISMA) guidelines^(24)^, using the three electronic databases PubMed (https://pubmed.ncbi.nlm.nih.gov/), Web of Science (https://clarivate.com/products/web-of-science/) and Embase (http://www.elsevier.com/online-tools/embase). The search was conducted independently by the two authors (MOK and OAG) in February 2022. A supplementary literature search on the 15 July 2022 did not identify any relevant articles published since the first search.

The following search terms were combined to identify relevant articles on the effects of fish or fish intake on serum/plasma cholesterol concentration in rodents, compared with a control group fed casein: ((fish) OR (fish protein hydrolysate) OR (marine) OR (anchovy) OR (bass) OR (billfish) OR (bonito) OR (capelin) OR (carp) OR (catfish) OR (cod) OR (eel) OR (flatfish) OR (haddock) OR (halibut) OR (herring) OR (mackerel) OR (menhaden) OR (pike) OR (plaice) OR (pollock) OR (pollack) OR (redfish) OR (saithe) OR (salmon) OR (sardine) OR (saury) OR (tilapia) OR (trout) OR (tuna) OR (turbot) OR (sprat) OR (whiting)) AND ((chinchilla) OR (guinea pig) OR (hamster) OR (mice) OR (mouse) OR (rat) OR (rodent)) AND ((cholesterol) OR (bile acid) OR (bile acids) OR (hypercholesterolemia) OR (LDL) OR (HDL)) AND (casein).

The reference lists of the reviewed articles were checked manually to identify relevant studies. We searched for reviews on the topic using PubMed, Web of Science and Embase, and we searched PROSPERO to avoid overlap with similar systematic reviews.

### Selection criteria

The search strategy was composed from the PICO concept (population, interventions, comparisons and outcomes) framework^(25)^ and was based on keywords for each of the PICO categories. Eligibility criteria were (1) population: only articles from intervention studies using rodents were included, but articles using neonate rodents, surgical animal models, rodents with chemically induced diseases or metabolic changes were excluded, (2) intervention: the intervention must comprise proteins from fish as part of a diet with regular protein content (14–25 wt%) for a period of more than 5 days, thus excluding proteins from marine sources other than fish, proteins administered by oral gavage or in drinking water, single-dose administration and short time studies (≤ 5 d), (3) comparison: the study design must include a control group fed casein as the sole dietary protein source, and the total protein content must be similar in the intervention diet and in the control diet for direct comparison and (4) outcomes: the main outcome was circulating TC concentration, the secondary outcomes were HDL-cholesterol, LDL-cholesterol, hepatic TC, daily faecal excretion of TC and bile acids, hepatic mRNA level/protein content/activity of CYP7A1, HMG-CoA reductase, LDL-receptor and SOAT2, and adiposity. Review articles, protocols, abstracts, posters and grey literature were not included. We did not exclude articles on the basis of the articles’ publication year or language during the identification process. Articles were manually removed if there were no English full texts available.

**Fig. 1.**
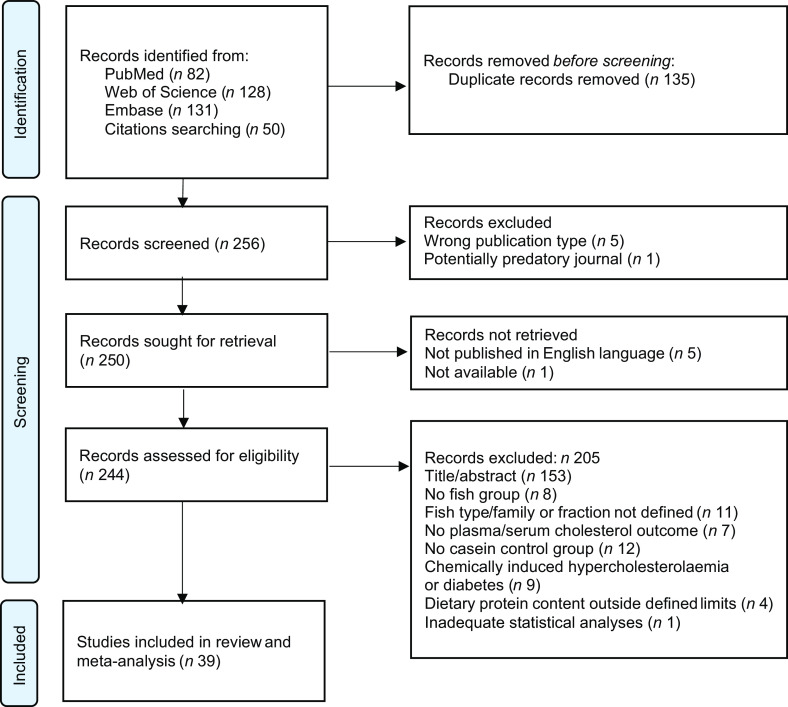
PRISMA flow chart of the literature search via databases, showing the selection of studies for inclusion in the systematic review and meta-analysis.

MOK and OAG individually performed the search and evaluated the articles. The retrieved articles from PubMed, Web of Science and Embase were collected in the free web-tool Rayyan (https://www.rayyan.ai) where duplicates were identified by the software and then removed manually. The screening was performed in two phases. The initial screening was based on the title and abstract, followed by a full-text screening of the eligible publications for final inclusion. In each phase, both authors independently assessed each article and any discrepancies were resolved through discussion, and articles were included or excluded based on the eligibility criteria. The PRISMA 2020 flow diagram^(24)^ gives an overview of the selection process (Fig. 1). In total, thirty-nine articles were found eligible and were included in this systematic review and meta-analysis.

### Data extraction and criteria appraisal

Data were extracted from article text, tables and figures based on the categories defined for each of the PICO categories described above. Data were extracted from the articles and included rodent species, strain, sex, bodyweight and age at baseline, experimental and control groups, number of animals per group, detailed description of the intervention diet (fish species, fish fraction, preparation of the fish, percentage of protein content from fish in the intervention diet), diet availability (e.g. *ad libitum* or pair feeding), the duration of the intervention period and the prandial state at the time of euthanisation. Data collected for outcome measurements included the main outcome, i.e. circulating TC concentration which was described in all included articles, and the secondary outcomes were HDL-cholesterol, LDL-cholesterol, hepatic TC content, daily faecal excretion of TC and bile acids, and hepatic mRNA level/protein content/activity of CYP7A1, HMG-CoA reductase, LDL-receptor and SOAT2. In addition, information on the adiposity at the endpoint, dietary intake and growth (bodyweight gain) were extracted.

The results were defined as statistically significant where *P* < 0·05. For articles that included multiple dietary groups, only data from the relevant fish and casein groups were extracted and are included in this review. Diet groups where fish was combined with other animal or plant proteins, oils or carbohydrates without an identical combination in the casein control group were not included, since the reported results could not be attributed to fish alone. The reviewed articles may therefore contain additional experimental groups or additional experiments that are not included in this review. The data extraction was completed independently by MOK and OAG. When inconsistencies were observed in the articles, e.g. missing data or information, or suspicion of possible errors, the corresponding authors were contacted by e-mail or through ResearchGate (https://www.researchgate.net). An evaluation of the extracted data revealed that study designs in the included articles were highly heterogeneous, therefore a descriptive approach in addition to a meta-analysis was performed.

### Assessments of risk of bias and study quality

All reviewed articles were assessed for risk of bias by using the SYRCLE’s risk of bias tool^(26)^, which is an adapted version of Cochrane’s risk of bias tool for clinical randomised trials^(27)^. The SYRCLE tool contains ten entries, with detailed signalling questions to help uncover potential bias. The questions were answered with Yes (low risk of bias), No (high risk of bias) or Unclear (unclear risk of bias). The quality of the included articles was evaluated by using a combination of the CAMARADES checklist^(28)^ and items from the ARRIVE 2.0 guidelines^(29)^. We selected these items to incorporate matters subjectively viewed as necessary for evaluating the overall quality of the studies, and we avoided items already covered by the SYRCLE’s risk of bias tool. The articles were scored with one point for reporting the required information and zero points when information is missing, with a total score of maximum 10 points, with 1–3 points regarded as low quality, 4–7 points regarded as medium quality and 8–10 points regarded as high quality. MOK and OAG independently assessed each article for risk of bias and study quality, and any discrepancies were resolved through discussion.

### Statistical analyses

The effect of consuming diets with proteins from fish on circulating TC concentration (the primary outcome) from all thirty-nine included articles was meta-analysed using Review Manager v. 5.4.1^(30)^ (provided by the Cochrane Collaboration), with relevant control diets containing casein as the sole protein source as the comparator. Two articles reported the effect of proteins from baked salmon muscles as 25 % of dietary protein on serum TC concentration from the same experiment^(31,32)^, however the finding was included only once in the meta-analyses. The mean and standard deviation for endpoint TC concentration and the number of rodents were recorded for intervention and comparator groups. Standard deviation was calculated from the standard error of the mean when not provided in the articles. In articles presenting the TC concentration using graphs, we did our best to estimate each group’s mean and spread. For studies with more than one intervention group sharing a control group for statistical comparisons, the number of rodents in the control group was divided by the number of intervention groups, while retaining the values for both mean and standard deviation as recommended by the Cochrane Handbook for Systematic Reviews of Interventions^(33)^. For TC concentrations presented as mg/dl, these were converted to mmol/l by multiplying with 0·02586. Data were treated as continuous measures, and the intervention and comparator groups were compared using the random effects inverse-variance model, and the statistical heterogeneity between studies was evaluated and is expressed as measures of Cochran’s Q (*χ* ^2^ test) and I^2^. Sensitivity analyses were conducted through a leave-one-out analysis to evaluate the effect of confounding factors on the robustness. The comparisons with the highest positive and negative effect sizes, and the studies with the highest risk of bias or lowest quality of evidence, were excluded sequentially from the meta-analysis. The publication bias was evaluated by using a funnel plot. Subgroup analyses were conducted for fish fractions (muscle, by-products), individual fish families (i.e. *Gadidae*, *Salmonidae* and *Clupeidae*), for total and partial replacement of casein with proteins from fish, and for diets with and without added cholesterol/cholate. The result from the main meta-analysis was visualised as a forest plot. *P* < 0·05 was considered statistically significant. The secondary outcomes are reviewed narratively.

## Results

### Search results and study characteristics

We identified 256 potentially relevant articles, and of these, twelve were excluded (five were the wrong publication type, one was published in a potentially predatory journal, five were not published in English language and one was not available). Of the 244 articles assessed for eligibility, 153 were excluded based on the title or abstract, and fifty-two were excluded due to ineligibility issues after full-text screening. In total, thirty-nine articles were eligible for inclusion in this systematic review and meta-analysis (Fig. 1), comprising a total of 512 and 423 rodents in the intervention and comparator groups, respectively. Of the reviewed articles, twenty-eight used rats and eleven used mice. Eligible articles using rodents other than rats and mice were not identified. The study characteristics are presented in Table 1 and Supplementary Table 1. The included studies were published between the years 1986 and 2021. The vast majority of the studies used male rodents; only two of the thirty-nine reviewed articles reported the use of female rodents, both were mice studies^(34,35)^, and one article did not state the sex of the mice^(36)^. None of the articles reported using both male and female rodents. All reviewed articles stated the age and/or bodyweight at the initiation of the intervention period. The age of the rodents at the start of the intervention ranged from 4 weeks to 15 weeks, i.e. periadolescent and young adults. The durations of the intervention periods were between 1 and 12 weeks, with 4 weeks being the most common duration of intervention. The included articles reported the number of animals in each experimental group, with one exception^(37)^, and the group sizes ranged from four to fourteen rodents.

**Table 1.** Study characteristics and outcomes

First author Year Reference number	Sex and rodent strain BW and age at intervention start	Description of the experimental groups, the number of animals in each group (*n*) and a brief description of experimental diets (proteins, simple carbohydrates, fats and added cholesterol and cholate, shown as wt%)	Total cholesterol (TC), HDL-cholesterol and LDL-cholesterol concentrations in circulation, daily faecal excretion of cholesterol and total bile acids and proteins (CYP7A1, HMG-CoA reductase, LDL-receptor, SOAT-2) in the liver in fish protein group(s) compared with casein group
Jacques H. 1986^(39)^	Male Sprague Dawley rats BW: 165–180 g Age: N/A	1. Casein (100 % of protein), *n* 8 2. Cod muscle proteins (100 % of protein), *n* 8 3. Casein (100 % of protein) + 1 % cholesterol and 0·5 % cholic acid, *n* 8 4. Cod muscle proteins (100 % of protein) + 1 % cholesterol and 0·5 % cholic acid, *n* 8 With: 15 % protein, 10 % corn oil, 0·12 % sucrose	Serum TC: NS for the cod group fed diet not enriched with cholesterol and cholate and lower for the cod group fed diet with added cholesterol and cholate Liver TC, HDL-cholesterol, LDL-cholesterol and faecal cholesterol and TBA, CYP7A1, HMG-CoA reductase, LDL-receptor, SOAT-2: N/A
Jacques H. 1988 ^(63)^	Male Sprague Dawley rats BW: 150- 175 g Age: N/A	1. Casein (100 % of protein), *n* 8 2. Cod muscle proteins (100 % of protein), *n* 8 With: 15 % protein, 10 % corn oil, 1 % cholesterol, 0·5 % cholic acid.	Plasma TC: Lower in the cod group Plasma HDL-cholesterol, LDL-cholesterol: NS Hepatic TC, faecal TC and TBA, CYP7A1, HMG-CoA reductase, LDL-receptor, SOAT-2: N/A
Hurley C. 1995^(66)^	Male Sprague Dawley rats BW: ∼ 230 g Age: N/A	1. Casein (100 % of protein) + corn starch 2. Casein (100 % of protein) + sucrose 3. Cod muscle proteins (100 % of protein) + corn starch 4. Cod muscle proteins (100 % of protein) + sucrose With: 20 % protein, 7 % coconut oil, 1 % corn oil, 59–60 % sucrose/corn starch, 1 % cholesterol Rats were fasted for 16 h (*n* 10 in each group) or were non-fasted (*n* 10 in each group).	Fasted state: Plasma TC and HDL-cholesterol: lower in cod groups (protein effect) Liver TC: NS (protein effect) Non-fasted state: Plasma TC and HDL-cholesterol: lower in the cod groups (protein effect) Liver TC: N/A Fasted and fed state: Plasma LDL-cholesterol, faecal TC and TBA, CYP7A1, HMG-CoA reductase, LDL-receptor, SOAT-2: N/A
Demonty I. 2003^(40)^	Male Sprague Dawley rats BW: ∼ 200 g Age: N/A	1. Casein (100 % of protein) with beef tallow, *n* 14 2. Casein (100 % of protein) with menhaden oil, *n* 14 3. Cod muscle proteins (100 % of protein) with beef tallow, *n* 14 4. Cod muscle proteins (100 % of protein) with menhaden oil, *n* 14 With: 20 % protein, 10 % beef tallow/menhaden oil, 4 % soya oil, 1 % cholesterol	Plasma and hepatic TC: lower for cod groups (protein effect) Plasma HDL-cholesterol and LDL-cholesterol, faecal TC and TBA, CYP7A1, HMG-CoA reductase, LDL-receptor, SOAT-2: N/A
Wergedahl H. 2004^(68)^	Experiment 1: Male Wistar rats BW: 85 ± 5 g Age: N/A Experiment 2: Male obese Zucker *fa/fa* rats BW: 120 ± 3 Age: N/A	Exp 1: 1. Casein (100 % of protein), *n* 6 2. Salmon muscle proteins (100 % of protein), *n* 6 With: 20 % protein, 10 % soya oil, 11 % sucrose Exp 2: 1. Casein (100 % of protein), *n* 6 2. Salmon muscle proteins (100 % of protein), *n* 6 With: 20 % protein, 10 % soya oil, 11 % sucrose	Exp 1: Plasma TC and HDL-cholesterol: NS Plasma LDL-cholesterol, liver TC, faecal TC and TBA, CYP7A1 and LDL-receptor: N/A Exp 2: Plasma TC and HDL-cholesterol: lower in the salmon group Hepatic activity of HMG-CoA reductase: higher in the salmon group Hepatic microsomal activity of SOAT-2: lower in the salmon group Plasma LDL-C, liver TC, faecal TC and TBA, CYP7A1 and LDL-receptor: N/A
Shukla A. 2006^(67)^	Exp. 1: Male Sprague Dawley rats BW: 72 ± 6 sd g Age: N/A Exp. 2: Male Sprague Dawley rats BW: 77 ± 5 g Age: N/A	Exp. 1: fasted 12h 1. Casein (100 % of protein), *n* 12 2. Pollock muscle proteins (100 % of protein), *n* 12 With: 19 % protein, 10 % lard, 20 % sucrose, 0·05 % cholesterol Exp. 2: not fasted 1. Casein (100 % of protein), *n* 12 2. Pollock muscle proteins (100 % of protein), *n* 12 With: 19 % protein, 10 % lard, 20 % sucrose, 0·05 % cholesterol	Exp. 1: fasted 12h Plasma TC and HDL-cholesterol: lower in the pollock group Plasma LDL-cholesterol: NS Liver TC: higher in the pollock group Faecal TC and TBA, CYP7A1, HMG-CoA reductase, LDL-receptor, SOAT-2: N/A Exp. 2: not fasted Plasma TC: NS Plasma LDL-cholesterol and liver TC: higher in the pollock group Plasma HDL-cholesterol: lower in the pollock group Faecal TBA, hepatic mRNA levels of HMG-CoA reductase and LDL-receptor: higher in pollock group Hepatic mRNA levels of CYP7A1, SOAT-2: NS Faecal TC: N/A
Moriya H. 2007^(36)^	C57BL/6J mice (sex N/A) BW: 25–30 g Age: 9 weeks	Pre-diet: 17 % lard 1. Casein (100 % of protein), *n* 6 2. Herring roe proteins (25 % of protein), *n* 6 With: 20 % protein, 7 % soya oil, 10 % sucrose	Plasma TC, HDL-cholesterol and LDL-cholesterol, liver TC: NS Faecal TC and TBA, CYP7A1, HMG-CoA reductase, LDL-receptor, SOAT-2: N/A
Hosomi R. 2009^(69)^	Male Wistar rats BW: 95–115 g Age: 5 weeks	1. Casein (100 % of protein), RGD, *n* 7 2. Pollock muscle proteins (50 % of protein), RGD, *n* 7 3. Casein (100 % of protein), RGD + CC, *n* 7 4. Pollock muscle proteins (50 % of protein), RGD + CC, *n* 7 5. Casein (100 % of protein), HFD, *n* 7 6. Pollock muscle proteins (50 % of protein), HFD, *n* 7 7. Casein (100 % of protein), HFD + CC, *n* 7 8. Pollock muscle proteins (50 % of protein), HFD + CC, *n* 7 RGD (regular diet): 20 % protein,7 % soya oil, 10 % sucrose HFD (high-fat diet: 20 % protein, 23 % lard, 7 % soya oil, 10 % sucrose CC: diets were added 0·5 % cholesterol and 0·1 % sodium cholate	RGD: Serum TC and LDL-cholesterol, liver TC: NS for the pollock group fed diet without added cholesterol, lower when fed CC-diet HDL-cholesterol: NS for both groups Faecal TC and TBA: N/A Hepatic mRNA level of CYP7A1: NS for the pollock group fed diet without added cholesterol, higher for the pollock group fed CC-diet Hepatic mRNA level of HMG-CoA reductase, LDL-receptor, SOAT-2: NS for both pollock groups (with and without added cholesterol) HFD: Serum TC and LDL-cholesterol, liver TC: NS for the pollock group fed diet without added cholesterol, lower when fed CC-diet HDL-cholesterol: NS for both groups Faecal TC and TBA: N/A Hepatic mRNA level of CYP7A1: NS for the pollock group fed diet without added cholesterol, higher for the pollock group fed CC-diet Hepatic mRNA level of HMG-CoA reductase, LDL-receptor, SOAT-2: NS for both pollock groups (with and without added cholesterol)
Liaset B. 2009^(49)^	Male Wistar rats BW: ∼ 60g Age: N/A	1. Casein (100 % of protein), *n* 5 2. Saithe frame proteins (100 % of protein), *n* 5 With: 20 % protein, 10 % soya oil, 9 % sucrose	Plasma TC and HDL-cholesterol: NS Faecal TBA: higher in the saithe group Hepatic mRNA level of CYP7A1: NS Plasma LDL-cholesterol, liver TC and faecal TC, HMG-CoA reductase, LDL-receptor, SOAT-2: N/A
Narayan B. 2009^(43)^	Male Wistar rats BW: 40 ± 2 g Age: 4 weeks	1. Casein (100 % of protein), *n* 6 2. Tuna muscle proteins (25 % of protein), *n* 6 With: 20 % protein, 7 % soya oil, 10 % sucrose	Plasma TC: lower in the tuna group Plasma HDL-cholesterol and LDL-cholesterol, liver TC: NS LDL-receptor: N/A Faecal TC and TBA: higher in tuna group Hepatic mRNA level of CYP7A1, HMG-CoA reductase, SOAT-2: NS
Jacques H. 2010^(38)^	Male Wistar rats BW: 80–85 g Age: N/A	1. Casein (100 % of protein), *n* 8 2. Cod muscle proteins (100 % of protein), *n* 7 With: 20 % protein, 10 % lard, 4 % soya oil, 21·8 % sucrose, 1 % cholesterol	Plasma TC: lower in the cod group Hepatic TC: NS Plasma HDL-cholesterol and LDL-cholesterol, faecal TC and TBA, CYP7A1, HMG-CoA reductase, LDL-receptor, SOAT-2: N/A
Mizushige T. 2010^(61)^	Male Sprague Dawley rats BW: ∼160 g Age: 6 weeks	1. Casein (100 % of protein), *n* 8 2. Pollock muscle proteins (100 % of protein), *n* 8 With: 20 % protein, 21 % lard, 3 % soya oil and 22 % sucrose	Serum TC and HDL-cholesterol, liver TC, faecal TBA: NS Serum LDL-cholesterol and faecal TC, CYP7A1, HMG-CoA reductase, LDL-receptor, SOAT-2: N/A
Hosomi R. 2010^(65)^	Male Wistar rats BW: ∼130g Age: 5–6 weeks	1. Casein (100 % of protein), *n* 7 2. Salmon protamine proteins (10 % of protein), *n* 7 3. Salmon protamine proteins (25 % of protein), *n* 7 With: 20 % protein, 7 % soybean oil, 10 % sucrose	Serum TC and LDL-cholesterol: NS for 10 %, lower for 25 % protamine group Serum HDL-cholesterol: NS in both groups Hepatic TC: lower in both groups Faecal TC: NS for 10 %, higher for 25 % Faecal TBA: higher for both groups Hepatic mRNA level of CYP7A1, HMG-CoA reductase, LDL-receptor: NS for both groups SOAT-2: N/A
Hosomi R 2011^(58)^	Male Wistar rats BW: N/A Age: 6 weeks	1. Casein (100 % of protein), *n* 7 2. Pollock muscle proteins (50 % of protein), *n* 7 With: 18 % protein 7 % soya oil, 10 % sucrose, 0·5 % cholesterol, 0·1 % sodium cholate	Serum TC and LDL-cholesterol, liver TC: lower in the pollock protein group Serum HDL-C: NS Faecal TC and TBA: higher in the pollock protein group CYP7A1, HMG-CoA reductase, LDL-receptor, SOAT-2: N/A
Liaset B. 2011^(55)^	Experiment 1: Male Wistar rats BW ∼ 60 g Age N/A Male Wistar rats BW N/A Age N/A	Exp 1: 1. Casein (100 % of protein), *n* 6 2. Salmon by-product proteins (100 % of protein), *n* 6 With: 21 % protein, 21 % lard, 3 % soya oil, 20 % sucrose Exp 2: 1. Casein (100 % of protein), *n* 5 2. Salmon by-product proteins (100 % of protein), *n* 5 With: 21 % protein, 21 % lard, 3 % soya oil, 20 % sucrose	Exp 1: Liver TC: lower in the salmon group Faecal TBA: NS Hepatic mRNA level of CYP7A1: NS Plasma TC, HDL-cholesterol and LDL-cholesterol, and faecal TC, HMG-CoA reductase, LDL-receptor, SOAT-2: N/A Exp 2: Plasma TC, HDL-cholesterol and LDL-cholesterol: NS Liver TC, faecal TC and TBA, CYP7A1, HMG-CoA reductase, LDL-receptor, SOAT-2: N/A
Louala S. 2011^(51)^	Male Wistar rats BW: 80 ± 5 g Age: N/A	Pre-diet: 1·5 % of cholesterol and 0·75 % cholic acid 1. Casein (100 % of protein), *n* 6 2. Sardine muscle proteins (100 % of protein), *n* 6 With: 20 % protein, 4 % sucrose, 5 % oil mixture (olive oil, nut oil and sunflower oil), 1·5 % cholesterol and 0·75 % cholic acid.	Serum TC and LDL-cholesterol hepatic TC: NS Faecal TC: lower in the sardine group Serum HDL-cholesterol, faecal TBA, CYP7A1, HMG-CoA reductase, LDL-receptor, SOAT-2: N/A
Bjørndal B 2012^(34)^	Female transgenic hTNF*α* mice BW: ∼ 20 g Age: 6–8 weeks	1. Casein (100 % of protein), *n 5* 2. Herring roe proteins (75 % of protein), *n 5* 3. Herring milt proteins (75 % of protein), *n 5* With: 20 % protein, 10 % sucrose, 2 % soya oil and 23 % lard in the casein diet, 19 % lard in the roe group and 22 % lard in the milt diet	Plasma TC and HDL-cholesterol: higher in the herring roe group, NS for the herring milt group Plasma LDL-cholesterol and liver TC: higher in both groups Hepatic mRNA level of CYP7A1: NS in the herring roe group, higher in the herring milt group Hepatic mRNA level of LDL-receptor: lower in both groups Faecal TC and TBA, HMG-CoA reductase, SOAT-2: N/A
Hosomi R. 2012^(59)^	Male Wistar rats BW: 95–115 g Age: 5 weeks	1. Casein (100 % of protein), *n* 7 2. Pollock muscle proteins (50 % of protein), *n* 7 3. Casein (100 % of protein) + 0·5 % cholesterol and 0·1 % sodium cholate, *n* 7 4. Pollock muscle proteins (50 % of protein) + 0·5 % cholesterol and 0·1 % sodium cholate, *n* 7 With: 18 % protein, 7 % soya oil, 10 % sucrose	Serum TC and LDL-cholesterol, liver TC: lower in the pollock protein groups with or without added cholesterol Serum HDL-cholesterol, faecal TC and TBA: higher in the pollock protein groups with or without added cholesterol Hepatic mRNA level of CYP7A1, HMG-CoA reductase, LDL-receptor, SOAT-2: NS
Hosomi R 2013^(57)^	Male Wistar rats BW: ∼ 130 g Age: 6 weeks	1. Casein (100 % of protein) and soya oil, *n* 7 2. Pollock muscle proteins (50 % of protein) and soya oil, *n* 7 3. Casein (100 % of protein) and tuna oil, *n* 7 4. Pollock muscle proteins (50 % of protein) and tuna oil, *n* 7 With: 20 % protein, 7 % soya oil or 5 % soya oil + 2 % tuna oil, and 10 % sucrose	Serum TC and LDL-cholesterol, liver TC: lower in the pollock groups (protein effect) Serum HDL-cholesterol, faecal TC and TBA: higher in the pollock groups (protein effect) Hepatic mRNA level of CYP7A1, HMG-CoA reductase, LDL-receptor: NS SOAT-2: N/A
Parolini C. 2014^(35)^	Female apoE^-/^-mice from the breeding strain C57BL/6, BW: N/A Age: 9 weeks	1. Casein (100 % of protein), *n 4* 2. Salmon spine proteins (21 % of protein), *n 4* With: 21 % protein, 21 % lard, 2 % soya oil and 12 % sucrose	Plasma TC: NS Hepatic activity of SOAT-2: NS Plasma HDL-cholesterol, LDL- cholesterol, liver TC, faecal TC and TBA, CYP7A1, HMG-CoA reductase, LDL-receptor: N/A
Kawabata F. 2015^(62)^	Male Sprague Dawley rats BW: ∼190 g Age: 6 weeks	1. Casein (100 % of protein), *n* 10 2. Pollock muscle proteins (100 % of protein), *n* 10 With: 17 % protein, 22 % lard, 3 % soya oil, 20 % sucrose	Serum TC and HDL-cholesterol, liver TC: NS Serum LDL-cholesterol, faecal TC and TBA, CYP7A1, HMG-CoA reductase, LDL-receptor, SOAT-2: N/A
Maeda H. 2015^(60)^	Male KK-*A* ^*y*^ mice BW: ∼ 18 g Age: 4 weeks	1. Casein (100 % of protein), *n* 8 2. Pollock muscle proteins (50 % of protein), *n* 8 With: 20 % protein, 13 % lard, 7 % soya oil, 10 % sucrose	Serum TC: NS Serum HDL-cholesterol and LDL-cholesterol, liver TC, and faecal TC and TBA, CYP7A1, HMG-CoA reductase, LDL-receptor, SOAT-2: N/A
Vik R. 2015^(50)^	Male C57BL/6J mice BW: ∼ 25–30 g Age: 10–11 weeks	1. Casein (100 % of protein), *n 8* 2. Salmon by-product proteins E1 (25 % of protein), *n 6* 3. Salmon by-product proteins E2 (25 % of protein), *n 6* 4. Salmon by-product proteins E4 (25 % of protein), *n 6* With: 20 % protein, 21 % lard and 3 % soya oil	Plasma TC and HDL-cholesterol: higher in the E2 salmon group, NS for E1 and E4 groups Plasma LDL-cholesterol: NS for all groups Hepatic mRNA level of HMG-CoA reductase: lower in E1 and E4 groups, NS in the E2 group. Liver TC, faecal TC and TBA, CYP7A1, LDL-receptor, SOAT-2: N/A
Hosomi R. 2015^(70)^	Male Wistar rats BW: ∼122g Age: 5–6 weeks	1. Casein (100 % of protein), *n* 7 2. Salmon protamine proteins (25 % of protein), *n* 7 With: 20 % protein, 7 % soybean oil, 10 % sucrose	Serum TC, liver TC: lower in the protamine group Faecal TC and TBA: higher in the protamine group Serum HDL-cholesterol and LDL-cholesterol, CYP7A1, HMG-CoA reductase, LDL-receptor, SOAT-2: N/A
Chevrier G. 2015^(37)^	Male LDLR^−/−^/ApoB^100/100^ mice BW: N/A Age: 8–9 weeks	1. Casein/casein hydrolysate (1:1) (100 % of protein) with corn oil 2. Casein/casein hydrolysate (1:1) (100 % of protein) with fish oil 3. Salmon muscle hydrolysate proteins (50 % of protein) with corn oil 4. Salmon muscle hydrolysate proteins (50 % of protein) with fish oil With: 22 % protein, 24 % lard, 16 % corn oil or 12 % corn oil + 4 % fish oil, 25 % sucrose The number of mice was not explicitly stated, but according to figures and tables, *n* 10–14 /group	Plasma TC: NS (protein effect) Plasma HDL-cholesterol and LDL-cholesterol, liver TC, and faecal TC and TBA, CYP7A1, HMG-CoA reductase, LDL-receptor, SOAT-2: N/A
Song S. 2016^(45)^	Male Sprague Dawley rats BW: 85–95 g Age: 4 weeks	1. Casein (100 % of protein), *n* 10 2. Carp muscle proteins (100 % of protein), *n* 10 With: 18 % protein, 7 % soya oil, 10 % sucrose	Plasma TC: lower in the carp group Liver TC: NS Plasma HDL-cholesterol and LDL-cholesterol, faecal TC and TBA, CYP7A1, HMG-CoA reductase, LDL-receptor, SOAT-2: N/A
Drotningsvik A. 2016^(42)^	Male Zucker fa/fa rats BW: 350 ± 20 g Age: N/A	1. Casein (100 % of protein), *n* 6 2. Herring by-product proteins (25 % of protein), *n* 6 3. Salmon by-product proteins (25 % of protein), *n* 6 With: 20 % protein, 7 % soya oil, 10 % sucrose	Serum TC: NS for both groups HDL-cholesterol and LDL-cholesterol: lower in the herring group, NS for the salmon group Liver TC and faecal TC and TBA: NS for both groups CYP7A1, HMG-CoA reductase, LDL-receptor, SOAT-2: N/A
Hosomi R. 2017^(54)^	Male Wistar rats Experiment 1: BW: 70–74 g Age: N/A Experiment 2: BW: 80–87 g Age: N/A	Exp 1: 1. Casein (100 % of protein), *n* 6 2. Pollock muscle proteins (100 % of protein), *n* 6 3. Tuna muscle proteins (100 % of protein), *n* 6 With: 23 % protein, 13 % lard, 7 % soya oil, 10 % sucrose Exp 2: 1. Casein (100 % of protein), *n* 6 2. Pollock muscle proteins (100 % of protein), *n* 6 3. Tuna muscle proteins (100 % of protein), *n* 6 With: 23 % protein, 13 % lard, 7 % soya oil, 10 % sucrose, 0·5 % cholesterol and 0·1 % sodium cholate	Exp 1: Serum TC and HDL-cholesterol: NS for both groups Liver TC: lower in the pollock protein group, NS for the tuna protein group Serum LDL-cholesterol, faecal TC and TBA: N/A Hepatic mRNA level of CYP7A1, HMG-CoA reductase, LDL-receptor, SOAT-2: NS for both groups Exp 2: Serum and liver TC: NS for the pollock group, higher in the tuna protein group Serum HDL-cholesterol, hepatic mRNA level of CYP7A1, HMG-CoA reductase, LDL-receptor, SOAT-2: NS for both groups Serum LDL-cholesterol, faecal TC and TBA: N/A
Vikøren L. 2017^(31)^	Male Zucker fa/fa rats BW: 350 ± 20 g Age: N/A	1. Casein (100 % of protein), *n* 5 2. Baked salmon muscle proteins (25 % of protein), *n* 6 3. Raw salmon muscle proteins (25 % of protein), *n* 6 With 20 % protein, 7 % soya oil, 10 % sucrose	Serum TC and LDL-cholesterol: lower in the baked salmon group, NS for the raw salmon group Serum HDL-cholesterol: lower for both groups Liver TC, faecal TC and TBA: NS for both groups CYP7A1, HMG-CoA reductase, LDL-receptor, SOAT-2: N/A
Maeda H. 2017^(56)^	Male KK-*A* ^*y*^ mice BW: ∼ 25 g Age: 4 weeks	1. Casein (100 % of protein), *n* 8 2. Tuna muscle proteins (50 % of protein), *n* 8 With: 20 % protein, 13 % lard, 7 % soya oil, 10 % sucrose	Serum TC: NS Serum HDL-cholesterol: higher in the tuna group Hepatic TC: lower in the tuna group Faecal TBA, hepatic mRNA level of CYP7A1, HMG-CoA reductase, LDL-receptor, SOAT-2: NS Serum LDL-C, faecal TC: N/A
Drotningsvik A. 2018^(44)^	Male Zucker fa/fa rats BW: 290 - 325 g Age: N/A	1. Casein (100 % of protein), *n* 6 2. Blue whiting (headed and gutted) proteins (33·3 % of protein), *n* 6 With: 20 % protein, 7 % soya oil, 10 % sucrose	Serum TC, HDL-cholesterol and LDL-cholesterol, liver TC: lower in the blue whiting group Faecal TC and TBA: NS Hepatic concentration of HMG-CoA reductase and LDL-receptor: lower in the blue whiting group CYP7A1 and SOAT-2: N/A
Vikøren L. 2018^(41)^	Male obese Zucker *fa/fa* rats BW: 355 g ± 10 sd Age: 8–9 weeks	1. Casein (100 % of protein), *n* 9 2. Cod muscle proteins (25 % of protein), *n* 10 With 20 % protein, 7 % soya oil, 10 % sucrose	Serum TC, HDL-cholesterol, LDL-cholesterol: lower in the cod group Liver TC, faecal TC and TBA, hepatic mRNA level of CYP7A1: NS Hepatic mRNA level of HMG-CoA reductase, LDL-receptor, SOAT-2: lower in the cod group
Vikøren L. 2018^(32)^	Experiment 1: Male Zucker fa/fa rats BW: 350 g ± 20 sd Age: 8–9 weeks Experiment 2: Male Long-Evans rats BW: 430 g ± 30 sd Age: 13–15 weeks	Experiment 1: 1. Casein (100 % of protein), *n* 5 2. Salmon muscle proteins (25 % of protein), *n* 6 With: 20 % protein, 7 % soya oil, 10 % sucrose Experiment 2: 1. Casein (100 % of protein), *n* 6 2. Salmon muscle proteins (25 % of protein), *n* 6 With: 20 % protein, 7 % soya oil, 10 % sucrose	Experiment 1: Serum TC, HDL-cholesterol, LDL-cholesterol: lower in the salmon group Liver TC, and faecal TC and TBA, CYP7A1, HMG-CoA reductase, LDL-receptor, SOAT-2: N/A Experiment 2: Serum TC, HDL-cholesterol, LDL-cholesterol: NS Liver TC, and faecal TC and TBA, CYP7A1, HMG-CoA reductase, LDL-receptor, SOAT-2: N/A
Affane F. 2018^(46)^	Male Wistar rats BW: 400 ± 20 g Age: N/A	1. Casein (100 % of protein), *n* 6 2. Sardine muscle proteins (100 % of protein), *n* 6 3. Sardine by-product proteins (100 % of protein), *n* 6 With: 19 % protein, 20 % mutton fat, 4 % sucrose	Serum TC and liver TC: lower in both groups Faecal TC: higher in both groups Serum HDL-cholesterol and LDL-cholesterol, faecal TBA, CYP7A1, HMG-CoA reductase, LDL-receptor, SOAT-2: N/A
Wang Y.W. 2019^(47)^	Male C57BL/6J mice BW: 40–45 g at start of intervention Age: 5 weeks at initiation of high-fat pre-diet, 11 weeks at start of intervention	Pre-diet: 32 % lard. 1. Casein (100 % of protein), *n* 12 2. Herring milt proteins (15 % of protein), *n* 12 3. Herring milt proteins (35 % of protein), *n* 12 4. Herring milt proteins (70 % of protein), *n* 12 With: 20 % protein, 28–32 % lard, 3 % soya oil, 9 % sucrose	Serum TC: lower in the 70 % milt protein hydrolysate group, NS for the 35 % and 15 % milt protein hydrolysate groups Serum HDL-cholesterol and LDL-cholesterol, liver TC, faecal TC and TBA, CYP7A1, HMG-CoA reductase, LDL-receptor, SOAT-2: N/A
Wang Y. 2020^(52)^	Male C57BL/6J mice BW: ca 40–45g Age: 12 weeks	1. Casein (100 % of protein), *n* 12 2. Herring milt dry protein powder (70 % of protein), *n* 12 3. Herring milt protein hydrolysate (70 % of protein), *n* 12 With: 20 % protein, 27–32 % lard, 3 % soya oil, 8–9 % sucrose	Serum TC: lower in the milt dry powder group, NS for the milt hydrolysate group Serum HDL-cholesterol: NS for both groups Serum LDL-cholesterol, hepatic TC, faecal TC and TBA, CYP7A1, HMG-CoA reductase, LDL-receptor, SOAT-2: N/A
Maeda H. 2020^(64)^	Male leptin-deficient *ob/ob* mice BW: ∼ 34 g Age: 5 weeks	1. Casein (100 % of protein), *n* 7 2. Pollock muscle proteins (100 % of protein), *n* 7 With: 20 % protein, 13 % lard, 7 % soybean oil, 10 % sucrose	Serum TC and hepatic TC: lower in the pollock group. Serum HDL-cholesterol: NS Faecal TBA: higher in the pollock group. Hepatic mRNA level of CYP7A1: higher in the pollock group Hepatic mRNA level of HMG-CoA reductase, LDL-receptor: NS Serum LDL-cholesterol, faecal TC, SOAT-2: N/A
Hosomi R. 2020^(48)^	Male Wistar rats BW: N/A Age: 5 weeks	1. Casein (100 % of protein), *n* 8 2. Pollock muscle proteins (100 % of protein), *n* 8 With: 18 % protein, 7 % soya oil, 10 % sucrose	Serum TC, HDL-cholesterol: NS Serum LDL-cholesterol, liver TC, faecal TC and TBA, CYP7A1, HMG-CoA reductase, LDL-receptor, SOAT-2: N/A
Mijiti 2021^(53)^	Male C57BL/6J mice BW: N/A Age 6–7 weeks	1. Casein (100 % of protein), *n* 8 2. Salmon protamine proteins (20 % of protein), *n* 8 With: 25 % protein, 4 % soybean oil, 30 % lard, 10 % sucrose	Serum TC and HDL-cholesterol: lower in the protamine group Liver TC: NS Faecal TC and TBA: higher in the protamine group Hepatic mRNA level of HMG-CoA reductase, LDL-receptor: higher in the protamine group Hepatic mRNA level of CYP7A1: NS Serum LDL-cholesterol, SOAT-2: N/A

CYP7A1, cholesterol 7 *α*-hydroxylase; N/A, data not available; NS, not statistically significant; SOAT-2, sterol *O*-acyltransferase 2; TBA, total bile acids; TC, total cholesterol.

The blood samples for analyses of TC, HDL-cholesterol and LDL-cholesterol were collected at the time of euthanasia for all articles except one, where blood samples were collected 1 week prior to euthanasia^(35)^. Blood was sampled from fasting rats in the majority of the articles^(31,32,34–53)^, while twelve articles declared that animals were not fasted^(54–65)^. Two articles presented results where half of the rats in each of the experimental groups were non-fasting while the rest of the animals were in a fasting state^(66,67)^, and four articles did not include information about the prandial state of the animals^(35,68–70)^. The details for prandial conditions at euthanasia are presented in online Supplementary Table 1.

Most of the included studies were designed with *ad libitum* access to feed^(31,32,34,36–46,48,50,53,54,56,59,62,63,65,66,68)^. The remaining studies were designed with pair feeding^(49,64)^, slightly restricted access to feed^(67)^ or gave no information on whether feed was freely available or controlled^(35,47,51,52,57,58,60,61,69,70)^. One article contained one study with *ad libitum* access to feed and one study with pair feeding^(55)^.

### Assessments of risk of bias and quality of evidence

The reviewed articles were assessed with the SYRCLE’s risk of bias tool as presented in Supplementary Table 2. The use of *sequence generation* (#1) for reducing selection bias was not reported in any of the reviewed articles, and all articles were therefore graded with ‘No’. Body weight and age were defined as the *baseline characteristics* (#2) that were compared between groups, and the majority of the articles used animals that were similar at baseline and were graded with ‘Yes’. Eight articles did include a baseline comparison of animals^(39,40,51,55,63,66–68)^, thus resulting in an unclear risk of bias. One article included experimental groups that differed in bodyweight at baseline, corresponding to 2–3 d of growth, possibly causing an increased risk of bias^(44)^. There was an unclear risk of bias in all studies for the *allocation concealment* (#3), thus all articles received ‘Unclear’ on this entry. Although none of the articles reported whether *random housing* (#4) was used, all articles received ‘Yes’, i.e. a low risk of bias, based on the signalling question ‘Is it unlikely that the outcome or the outcome measurement was influenced by not randomly housing the animals?’. Only one article^(41)^ reported the use of *blinding* in relation to *performance bias* (#5) and *detection bias* (#7). For the *random outcome assessment* (#6), we graded all articles as having an unclear risk of bias. When scoring the articles for *incomplete outcome data* (#8), most articles received ‘Yes’, i.e. a low risk of bias, as all the expected data were included or any missing data were accounted for. Ten articles received ‘No’, as they did not disclose why animals were missing from the reported outcomes^(35,37–39,47,49,50,52,63,66)^ and three studies did not disclose the number of animals in each assessment^(40,55,69)^ and therefore received grading as ‘Unclear’. For the last two entries; *selective outcome reporting* (#9) and *other sources of bias* (#10), we graded all articles as having an unclear risk of bias. To summarise, the included articles were scored with one or two ‘No’ and between one and five ‘Yes’, with the rest of the scores being ‘Unclear’. Since few of the entries in the SYRCLE’s tool were addressed, we conclude that the risk of bias was unclear for the included articles.

All included articles were assessed for the quality of evidence for the primary outcomes (online Supplementary Table 3). All articles were peer reviewed (#1), included information on the animal model and strain used (#2) and gave details on the statistical method (#7) and descriptive statistics with a measure of variability (#8) for the serum/plasma TC concentration. All articles except one^(36)^ stated the sex of the experimental animals (#3). Only five articles^(31,32,41,42,44)^ provided information about husbandry conditions and actions to improve animal welfare of the experimental animals (#4). For item #5, i.e. description of the procedures, we checked for information on when the blood was sampled, any use of anaesthesia when blood was sampled for TC analysis and if information regarding the prandial condition of the animals were provided. All articles described when blood was collected for TC analysis, but three articles did not provide information on prandial status^(68–70)^ and one article gave no information regarding any use of anaesthesia at the time of sampling^(55)^. Eighteen articles described the analysis of serum/plasma TC, including the name and brand of assays and kits (#6), while the other articles referred to the laboratory that conducted the analysis or the instrument that was used. Compliance with animal welfare regulations (#9) was declared in all articles except the two oldest; Jacques *et al.* 1986^(39)^ and Jacques *et al.* 1988^(63)^. Statement of potential conflict of interests (#10) was not declared in twenty-one articles. The overall quality was high for the included articles, with a mean score of 8 (range 6–10).

### Details on the rodent models

The included papers used the following rat models: Wistar rats^(38,43,46,48,49,51,54,55,57–59,65,69,70)^, Sprague Dawley rats^(39,40,45,61–63,66,67)^ or obese Zucker *fa/fa* rats^(31,41,42,44)^ or combined studies in Zucker *fa/fa* rats with Wistar rats^(68)^ or Long-Evans rats^(32)^ (Table 1). Several mouse models were utilised: C57BL/6J mice^(36,47,50,52,53)^, apolipoprotein E null mice (apoE^-/^-, established in strain C57BL/6)^(35)^, ApoB100 only, LDL receptor knockout male mice (LDLR^-/^-/ApoB^100/100^)^(37)^, transgenic mice expressing human tumour necrosis factor-*α* (hTNF*α*, established in strain C57BL/6)^(34)^, leptin-deficient *ob/ob* mice^(64)^ and KK-*A* ^*y*^ obese mice^(56,60)^.

The Wistar rat, the Sprague Dawley rat, the Long-Evans rat and the C57BL/6J mouse are regarded as being normocholesterolemic when fed a regular diet. The obese Zucker *fa/fa* rat, which is one of the most widely used rat models for studies of metabolic complications and for possible treatments of obesity in humans, is hyperphagic and spontaneously develops metabolic abnormalities including elevated concentrations of serum LDL-, HDL- and VLDL-cholesterol^(71)^ and has impaired bile secretory function^(72)^. The apolipoprotein E null mouse^(73)^ and the LDLR-deficient mouse^(74)^ are hypercholesterolemic. The KK mouse carrying the yellow obese gene *A* ^*y*^ (KK-*A* ^*y*^) is obese and develops type 2 diabetes^(75)^. The leptin-deficient *ob/ob* mouse is obese, has elevated cholesterol in circulation and impaired transport of cholesterol from circulation to faeces^(76)^. The transgenic hTNF*α* mouse experiences metabolic alterations induced by inflammation^(77)^ but does not have elevated serum cholesterol concentration^(78)^.

### Details on the designs of the diets

All reviewed articles included a control group that was fed a diet with casein as the sole protein source for direct comparison of the intervention fish protein source. The dietary protein content was similar in the control (casein) diet and the fish intervention diet for all included studies. The dietary total protein content was between 15 and 25 wt% for all described experiments. All studies used semi-purified diets, i.e. diets combining a fish protein source or casein with pure ingredients or chemicals with a varying degree of refinement.

The reviewed articles show great heterogeneity regarding both the fish species investigated, and which parts of the fish that were used and how these were processed. Nine varieties of fish were explored, and the proteins from fish replaced between 15 and 100 % of the casein in the experimental diets, as detailed in Table 1. Articles used muscles from cod that were defatted^(38–40,63,66)^ or lyophilised^(41)^, muscles from pollock that were defatted^(48,54,57,58,60–62,64,67,69)^ or defatted and hydrolysed^(59)^ and frames from saithe^(49)^ or water-soluble (press liquid) meal from headed and gutted blue whiting^(44)^. Also, hydrolysed muscles^(37,68)^, lyophilised muscles^(31,32)^, hydrolysed by-products^(35,42,50,55)^ and protamine (from milt)^(53,65,70)^ from salmon were investigated. From herring, hydrolysed by-products^(42)^, spray-dried roe and milt^(34)^, defatted roe protein^(36)^, milt protein hydrolysate^(47)^ and dried or hydrolysed milt^(52)^ were tested. Studies were also conducted using defatted muscles^(51)^, defatted and hydrolysed muscles^(46)^, defatted and hydrolysed by-products^(46)^ from sardine, defatted muscles from tuna^(43,54,56)^ and defatted muscles from carp^(45)^.

The proteins from fish were included in a variety of diet designs, including diets with regular contents of fat and simple carbohydrates (≤ 7 wt% fat and ≤ 10 wt% of simple carbohydrates)^(31,32,36,41–45,48,51,57–59,65,69,70)^, high-fat diets (≥ 30 wt% fat) with regular content of simple carbohydrates (≤ 10 wt%)^(47,52,53,69)^, high-fat diets with moderately high content of simple carbohydrates (11–54 wt%)^(37)^, diets with moderately high-fat content (8–29 wt%) and regular content of simple carbohydrates (≤ 10 wt%)^(34,39,40,46,49,50,54,56,60,63,64)^, diets with moderately high-fat content and moderately high content of simple carbohydrates^(35,38,55,61,62,67,68)^ and diets with moderately high-fat content (10–30 %) and high content of simple carbohydrates (≥ 55 wt%)^(66)^. In some of the articles, cholesterol^(38,40,66,67)^ or a combination of cholesterol and cholate^(39,51,54,58,59,63,69)^ was added to the diets to promote elevation in the circulating cholesterol concentration. Cholate reduces the cholesterol catabolism in the liver by inhibiting the production of bile acids through down-regulation of hepatic CYP7A1 activity. None of the studies added cholesterol in order to balance the cholesterol content between the diets.

### Cholesterol metabolism

All reviewed articles describe the effects of fish or fish protein intake, i.e. from muscles or by-products, on serum/plasma TC concentration measured at the endpoint. In addition, many articles describe the effects on HDL-cholesterol, LDL-cholesterol and hepatic TC concentration, the daily faecal excretion of TC and bile acids and a selection of enzymes relevant to cholesterol metabolism, as detailed in Table 1. Only in one article was the circulating TC concentration measured at baseline after rats were fed a cholesterologenic pre-diet to induce hypercholesterolaemia, but statistical testing to compare TC concentrations at baseline and endpoint was not conducted^(51)^. Below we present the findings reported in the reviewed articles, organised by family of the fish sources.

#### Cod (family Gadidae)

A lower serum/plasma TC concentration was reported in Wistar rats^(38)^ and Sprague Dawley rats^(39,40,63,66)^ fed cod muscles as the sole protein source, but only when the diets were enriched with cholesterol alone or in combination with cholate. A lower HDL-cholesterol concentration was reported when cod muscle proteins were added to a high-carbohydrate diet^(66)^, whereas the HDL-cholesterol concentration was similar to the control group when rats were fed a diet with regular carbohydrate content^(63)^. The LDL-cholesterol concentration was measured in one of these studies, showing a similar concentration to that of the control group^(63)^, whereas the hepatic TC content was not affected^(38,66)^.

A lower concentration of circulating TC was reported in obese Zucker *fa/fa* rats fed 25 % cod muscle proteins as part of a regular diet, and this was accompanied by lower HDL-cholesterol and LDL-cholesterol concentrations and lower hepatic mRNA expressions of HMG-CoA reductase, LDL-receptor and SOAT-2 when compared with the control group^(41)^. The hepatic TC concentration, the faecal excretion of cholesterol and bile acids, as well as the CYP7A1 mRNA level were similar in rats fed cod muscle and control rats^(41)^.

#### Pollock (family Gadidae)

In several studies, the serum TC concentration was lower in Wistar rats fed pollock muscle protein as 50 % of total protein in diets with^(58,59,69)^ or without^(57,59)^ added cholesterol/cholate when compared with the respective control groups. This was accompanied by a lower LDL-cholesterol concentration^(57–59,69)^, a lower hepatic TC concentration^(57–59,69)^, a HDL-cholesterol concentration that was higher^(57,59)^ or similar^(58,69)^, a higher faecal excretion of TC and bile acids^(57–59)^ and a hepatic CYP7A1 mRNA level that was similar^(57,59)^ or higher^(69)^ to that of the corresponding control group. The gene expressions of HMG-CoA reductase^(57,59,69)^, LDL-receptor^(57,59,69)^ and SOAT-2^(59,69)^ were similar to those of the corresponding controls.

In other studies, the serum TC concentration was not affected in Wistar and Sprague Dawley rats fed diets with pollock muscle proteins as the sole protein source^(48,61,62)^ or as 50 % of protein^(69)^ in diets with no added cholesterol. In these studies, the LDL-cholesterol concentration^(69)^, the HDL-cholesterol concentration^(48,54,61,62,69)^, the liver TC concentration^(61,62,69)^ and the faecal excretion of bile acids^(61)^ were not affected. The serum TC concentration was also not affected in Wistar rats fed a diet with 100 % of protein from pollock muscle with either added or no added cholesterol and cholate^(54)^. However, the hepatic TC concentration was lower in rats fed a diet without added cholesterol and cholate but was not affected when the diet was enriched with cholesterol and cholate^(54)^, and the pollock diets did not affect the hepatic mRNA levels of CYP7A1, HMG-CoA reductase, LDL-receptor and SOAT-2^(54)^.

In a separate study, Sprague Dawley rats fed pollock muscle proteins as the sole protein source in diets enriched with cholesterol, the plasma TC concentration was lower compared with the control group in fasted rats, but was similar to controls when rats were in a non-fasted state^(67)^. Both fasted and non-fasted rats had a lower HDL-cholesterol concentration and a higher hepatic TC content, whereas the LDL-cholesterol concentration was higher in non-fasted rats but was not affected in fasted rats^(67)^. These unexpected differences were not commented upon in the article. Hepatic mRNA was assessed in the fasted rats and showed higher levels of HMG-CoA reductase and LDL-receptor in the pollock groups compared with controls, with no differences between the groups for the faecal excretion of bile acids or the CYP7A1 and SOAT-2 mRNA levels^(67)^.

Leptin-deficient *ob/ob* mice fed pollock muscles as the sole dietary protein source had a lower serum TC concentration, with lower hepatic TC concentration, higher faecal bile acid excretion and hepatic mRNA level of CYP7A1 compared with the casein group, whereas the HDL-cholesterol concentration and the gene expressions of HMG-CoA reductase and LDL-receptor were not affected^(64)^. A study in KK-*A* ^*y*^ mice found no effect of pollock muscle protein intake (50 % of dietary protein) on the circulating TC concentration^(60)^.

#### Saithe (family Gadidae)

One article investigated the effect of saithe on the circulating TC concentration by using hydrolysed saithe frames^(49)^. When Wistar rats were fed a regular diet with saithe frame proteins as the sole protein source, the plasma TC and HDL-cholesterol concentrations and the hepatic mRNA level of CYP7A1 were similar to those of the control group, whereas the faecal excretion of bile acids was higher compared to controls^(49)^.

#### Blue whiting (family Gadidae)

The effect of blue whiting on the circulating TC concentration was investigated in one article, using the water-soluble fraction from headed and gutted blue whiting as one-third of the dietary protein in a regular diet^(44)^. When fed to obese Zucker *fa/fa* rats, this diet resulted in lower serum concentrations of TC, HDL-cholesterol and LDL-cholesterol, a lower hepatic TC content and lower hepatic concentrations of HMG-CoA reductase and LDL-receptor, without affecting the faecal excretion of TC and bile acids^(44)^.

#### Salmon (family Salmonidae)

The serum TC and LDL-cholesterol concentrations were lower in obese Zucker *fa/fa* rats fed baked salmon muscles as 25 % of dietary protein in a regular diet^(31,32)^, whereas the equivalent amount of raw salmon muscle proteins did not affect serum TC and LDL-cholesterol concentrations in these rats^(31)^. Both the baked and the raw salmon diets led to a lower HDL-cholesterol concentration in the Zucker *fa/fa* rats, but neither diet affected the liver TC concentration or the faecal excretion of TC and bile acids^(31,32)^. When Long-Evans rats were fed 25 % of dietary protein from baked salmon, no effects were seen on the serum concentrations of TC, HDL-cholesterol and LDL-cholesterol^(32)^. Consuming a diet with salmon muscle hydrolysate as the sole protein source resulted in lower plasma TC and HDL-cholesterol concentrations in obese Zucker *fa/fa* rats, but concentrations were not affected in Wistar rats^(68)^. The activity of HMG-CoA reductase was higher, and the activity of SOAT-2 was lower, in the liver of Zucker *fa/fa* rats fed salmon muscle hydrolysate^(68)^.

A diet with hydrolysed by-products from salmon as the sole protein source did not affect the plasma concentrations of TC, HDL-cholesterol or LDL-cholesterol or the faecal excretion of bile acids in Wistar rats, but led to a lower hepatic TC content and a lower hepatic CYP7A1 mRNA level^(55)^. In obese Zucker *fa/fa* rats fed a diet with 25 % of proteins from hydrolysed salmon by-products, the serum TC, HDL-cholesterol and LDL-cholesterol concentrations, as well as the hepatic TC content and the faecal excretion of TC and bile acids, were not affected^(42)^. When three salmon by-product hydrolysates; E1 (spines, Acid protease A), E2 (spines, Umamizyme), and E4 (backbones and heads, Alcalase) constituted 25 % of protein in diets that were administered to C57BL/6J mice, the plasma TC and HDL-cholesterol concentrations were elevated in the E2 group, with no difference E1 and E4 groups when compared with the control group^(50)^. The LDL-cholesterol concentration was not affected in any of the groups, but the hepatic mRNA level of HMG-CoA reductase was lower in mice fed E1 and E4, and similar to controls in the E2 group^(50)^. Salmon by-product proteins as 21 % of dietary protein administered to apoE^-/^- mice^(35)^, and hydrolysed salmon muscle proteins as 50 % of dietary protein administered to LDLR^−/−^/ApoB^100/100^ mice^(37)^ did not affect the plasma TC concentration^(35)^.

Salmon protamine was tested in doses of 10 %^(65)^ and 25 %^(65,70)^ of protein in regular diets in Wistar rats. The lowest dose (10 %) did not affect the serum TC, HDL-cholesterol or LDL-cholesterol concentrations or the faecal TC excretion, but resulted in a lower hepatic TC content and a higher faecal bile acid excretion^(65)^. Rats fed diets with 25 % of proteins from protamine had lower serum concentrations of TC and LDL-cholesterol, a lower hepatic TC content and higher excretions of TC and bile acids, whereas the HDL-cholesterol concentration and the gene expressions of CYP7A1, HMG-CoA reductase and LDL-receptor in the liver were similar to controls^(65,70)^. C57BL/6J mice fed a diet with 20 % of protein from protamine in a high-fat diet had lower serum TC and HDL-cholesterol concentrations, higher faecal excretions of TC and bile acids, as well as elevated mRNA levels of HMG-CoA reductase and LDL-receptor compared with controls, but the hepatic TC content and the CYP7A1 gene expressions in liver were not affected^(53)^.

#### Herring (family Clupeidae)

Hydrolysed herring by-products, i.e. a mixture of heads, guts and backbones, did not affect serum and hepatic TC concentrations or the faecal excretion of TC and bile acids, but resulted in lower concentrations of both HDL-cholesterol and LDL-cholesterol in obese Zucker *fa/fa* rats when constituting 25 % of the protein in a regular diet^(42)^.

Herring milt protein hydrolysate caused a lower serum TC concentration when fed to C57BL/6J mice as 70 % of dietary protein in a high-fat diet, but did not affect the serum TC concentration in doses of 15 or 35 %^(47)^. Another study in C57BL/6J mice high-fat diets added herring milt found lower serum TC concentration in those fed 70 % herring milt dry powder, but not in those fed herring milt protein hydrolysate as 70 % of dietary protein, with no effect on HDL-cholesterol concentration in either group^(52)^. When transgenic hTNF*α* mice were fed a diet with moderately high-fat content and 75 % of protein as herring milt, no effects were seen on the plasma concentrations of TC and HDL-cholesterol, but the LDL-cholesterol concentration, the hepatic TC content and the hepatic CYP7A1 gene expression were higher, and the LDL-receptor mRNA level was lower compared with the control group^(34)^.

A regular diet with 25 % of protein as herring roe did not affect the plasma TC, HDL-cholesterol or LDL-cholesterol concentrations or the hepatic TC content in C57BL/6J mice^(36)^. In transgenic hTNF*α* mice, a moderately high-fat diet with 75 % of protein as herring roe resulted in higher serum TC, HDL-cholesterol and LDL-cholesterol concentrations, a higher hepatic TC content and a lower LDL-receptor mRNA level in the liver, while the CYP7A1 mRNA level was similar to the control group^(34)^.

#### Sardine (family Clupeidae)

The serum TC concentration was lower in Wister rats fed diets with moderately high-fat content with hydrolysed muscles or hydrolysed by-products (viscera, heads, skin and edges) from sardine as the sole protein source without added cholesterol^(46)^. Concomitant with this, both sardine groups had lower hepatic TC concentration and higher faecal TC excretion^(46)^. When Wistar rats were fed sardine muscles as the only protein source as part of a diet with regular fat content with added cholesterol and cholate, the serum TC and LDL-cholesterol concentrations as well as the hepatic TC content were not affected, whereas the faecal TC excretion was lower^(51)^.

#### Tuna (family Scombriadae)

A lower plasma TC concentration was reported in Wistar rats fed tuna muscle protein as 25 % of dietary protein in a regular diet, concomitant with a higher faecal excretion of TC and bile acids compared with the control group, but with no effects on the concentrations of HDL-cholesterol and LDL-cholesterol, the hepatic TC content, and the hepatic mRNA levels of CYP7A1, HMG-CoA reductase and SOAT-2^(43)^.

Higher serum and hepatic TC concentrations were observed in Wistar rats fed tuna muscle protein as the sole dietary protein in diets with moderately high fat content with added cholesterol and cholate, but were not affected in diets with no added cholesterol/cholate^(54)^. The HDL-cholesterol concentration and the gene expressions of CYP7A1, HMG-CoA reductase, LDL-receptor and SOAT-2 in the liver were not affected by tuna muscles in diets with or without added cholesterol^(54)^.

When KK-*A* ^*y*^ mice were fed a diet where tuna muscle protein constituted half of the protein in a moderately high-fat diet with no added cholesterol, the serum TC concentration, the faecal bile acid excretion and the gene expressions of CYP7A1, HMG-CoA reductase, LDL-receptor and SOAT-2 in the liver were similar to controls^(56)^. Nevertheless, these mice had a higher HDL-cholesterol concentration and a lower hepatic TC concentration^(56)^.

#### Carp (family Cyprinidae)

Carp was tested in one article by using dorsal muscles as the sole protein source in a regular diet^(45)^. When fed to Sprague Dawley rats, this diet resulted in a lower plasma TC concentration but did not affect the hepatic TC content.

### Meta-analyses

**Fig. 2.**
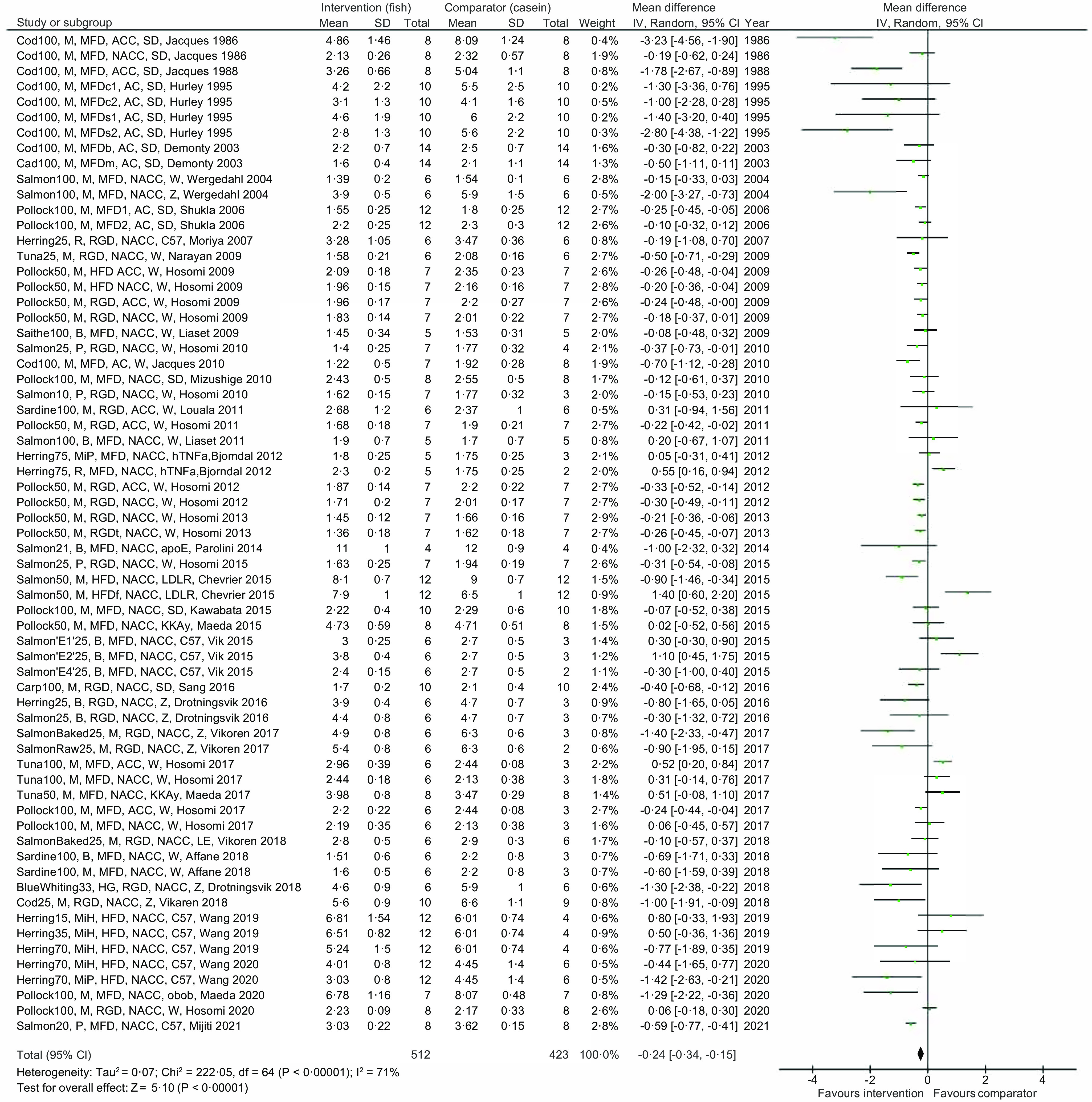
Meta-analysis using a random effects model presenting the effects of intake of proteins from fish muscles or fish residuals on circulating total cholesterol concentration (mmol/l) as a forest plot. The studies are described as [type of fish and dose], [fish fraction], [description of diet], [w/o cholesterol and cholate], [rodent strain], [first author and year of publication]. Type of fish and dose: the dose of fish protein is shown as percent of total dietary content; E1, spine hydrolysed with Acid Protease A; E2, spine hydrolysed with Umamizyme; E4, backbone and heads hydrolysed with Alcalase fish fraction; B, by-products; M, muscle; MiH, hydrolysed milt; MiP, dried milt powder; P, protamine; R, roe. Description of diet: HFD, high-fat diet; HG, headed and gutted; MFD, medium-fat diet; RGD, regular diet; s, high sucrose content; c, high corn starch content; b, added beef tallow; m, added menhaden oil; f, added fish oil; t, added tuna oil; 1, animals were fasted at euthanisation; 2, animals were nonfasted at euthanisation with or without enrichment with cholesterol and cholate: NACC, diet is not added cholesterol or cholate; AC, diet is added cholesterol; ACC, diet is added cholesterol and cholate. Rodent strain: apoE, apolipoprotein E null mice; C57, C57BL/6J mice; hTNFa, transgenic hTNF*α* mice; KKAy, KK-A^y^ mice; LDLR, ApoB100 only LDL-receptor knockout mice; LE, Long-Evans rats; obob, leptin-deficient ob/ob mice; sd, Sprague Dawley rats; W, Wistar rats; Z, Zucker fa/fa rats.

The main meta-analysis of differences in endpoint serum/plasma TC concentration in the intervention and comparator groups includes data from all thirty-nine reviewed articles, representing findings in 512 rodents in the intervention groups and 423 rodents in the comparator groups (Fig. 2). The meta-analysis revealed that diets containing proteins from fish resulted in lower circulating TC concentration in rodents compared with their respective control group, with a mean difference −0·24 mmol/l and 95 % confidence interval −0·34, −0·15 mmol/l, with an overall test for effect Z = 5·10 and *P* < 0·00001. When testing for statistical heterogeneity, *χ* ^2^ was 222·05 (*P* < 0·00001) and I^2^ was 71 %, thus reflecting that both the direction of the between-group differences and the magnitude of effect were highly heterogeneous in our meta-analysis.

**Fig. 3.**
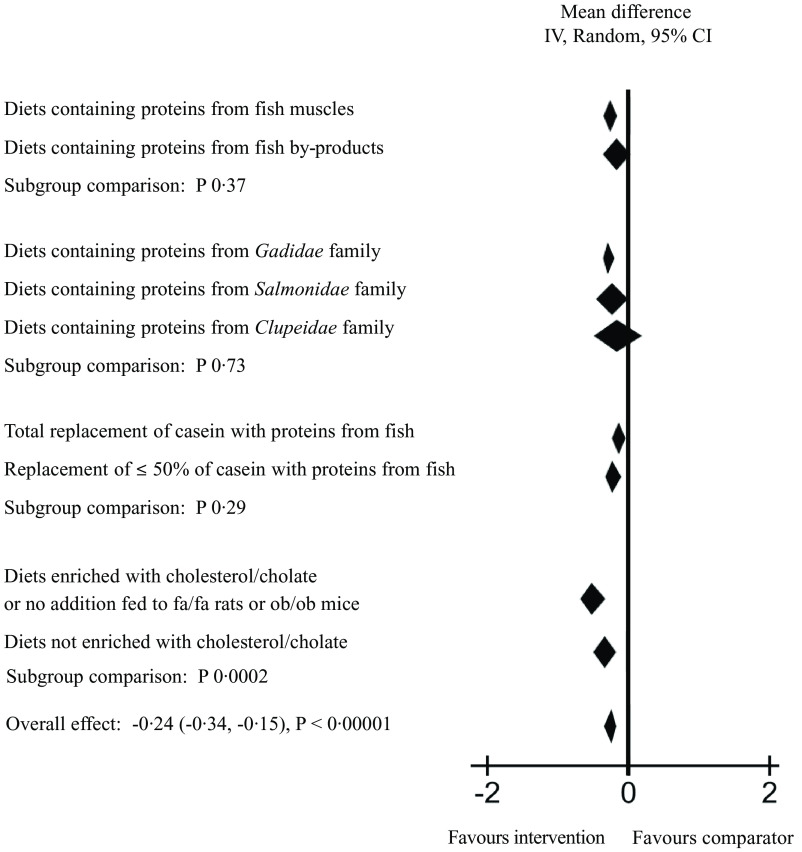
Subgroup analyses for meta-analysis using a random effects model presenting the effects of intake of proteins from fish muscles or fish residuals on circulating total cholesterol concentration (mmol/l) as a forest plot. We refer to online Supplementary Table 4 for further details on heterogeneity, effect and statistical significance.

To further explore the heterogeneity in the main meta-analysis, a series of subgroup analyses were conducted (Fig. 3 and online Supplementary Table 4). First, diets containing proteins from fish muscles or fish by-products were tested individually, and these meta-analyses gave a mean difference (95 % CI) of –0·27 (–0·37, 0·17) mmol/l for serum/plasma TC concentration for fish muscle proteins and -0·16 (-0·38, 0·06) mmol/l for fish by-product proteins, with *P* < 0·00001 (Z = 5·16) and *P* 0·16 (Z = 1·40), respectively. The heterogeneity was still high for both analyses, with Chi^2^ = 149·51 (*P* < 0·00001) and I^2^ = 72 % for fish muscle proteins and Chi^2^ = 72·47 (*P* < 0·00001) and I^2^ = 71 % for fish by-product proteins. When the subgroups were compared, no significant difference was detected between the effect of fish muscles and fish by-products on circulating TC concentration (*P* 0·37).

Next, subgroup analyses by fish family were conducted, including both fish muscle proteins and fish by-product proteins combined to investigate the effect on circulating TC concentration. The subgroup of rodents fed proteins from the *Gadidae* family (cod, pollock, saithe and blue whiting) were the largest contributors to the main meta-analysis, with a total of 514 rodents included in the analysis. The difference between the intervention and comparator groups was significant (*P* < 0·00001), with a mean difference of −0·28 (95 % CI; −0·38, −0·19) mmol/l and I^2^ of 60 %. For the subgroup fed proteins from the *Salmonidae* family (205 rodents included), the test for overall effect was borderline significant with P 0·05 and a mean difference of −0·24 (–0·48, 0·00) mmol/l, whereas the subgroups fed dietary proteins from the *Clupeidae* family (sardine and herring, a total of 150 rodents included) did not affect circulating TC concentration (P 0·44 and mean difference −0·14 (–0·50, 0·22) mmol/l). Subgroup analyses were not conducted for the groups fed proteins from the *Scombriadae* (tuna) and *Cyprinidae* (carp) families due to their small sample sizes. The comparison of the effect of *Gadidae, Salmonidae* and *Clupeidae* families on circulating TC concentration showed no significant differences between the subgroups (*P* 0·73).

A subgroup analysis was conducted to investigate whether a total replacement of casein or a replacement of 50 % or less of casein with proteins from fish had different impact on the circulating TC concentration. Both total and partial replacement with proteins from fish resulted in significantly lower serum/plasma TC concentration (mean difference −0·32 (–0·49, −0·15) with P 0·0002, and −0·24 (-0·35, −0·13) with *P* < 0·0001, respectively), with no significant difference between the subgroups (*P* 0·29).

We expected that the circulating TC concentration would increase over time in especially two subcategories; the genetically obese rodents which spontaneously develop hypercholesterolaemia after birth (the leptin-resistant Zucker *fa/fa* rats and the leptin-deficient *ob/ob* mice) and rodents fed diets enriched with cholesterol alone or in combination with cholate. A subgroup analysis of studies using these rodents *v*. the rest of the included studies revealed that both subgroups had significantly lower mean differences in serum/plasma TC concentration when intervention and comparator groups were compared; for the subgroup comprising *fa/fa* rats, *ob/ob* mice and rodents fed diets enriched with cholesterol/cholate, the mean difference was −0·53 (–0·72, −0·35) with *P* < 0·00001, and for the subgroup consisting of the remaining groups the mean difference was −0·13 (-0·23, −0·02) with P 0·02. The difference between the subgroups was significant (*P* 0·0002), with the strongest effect seen in the former subgroup.

The sensitivity analysis, conducted through a leave-one-out analysis of studies with the highest positive and negative effect sizes, and the studies with the highest risk of bias or lowest quality of evidence were excluded sequentially from the meta-analysis, did not alter the outcome measure (data not presented).

**Fig. 4.**
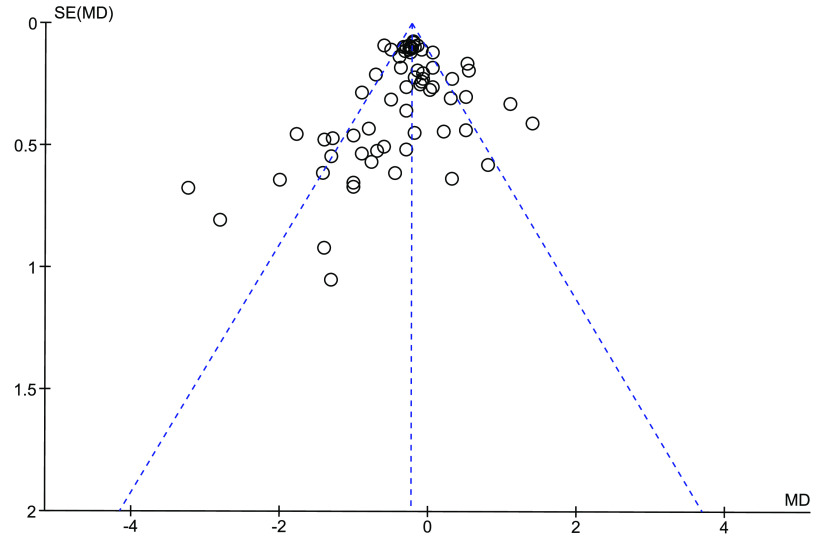
Funnel plot showing the effect estimate with 95 % CI for the effect of intake of diets containing proteins from fish on circulating total cholesterol concentration.

The asymmetry of the funnel plot (Fig. 4) was visually inspected to assess the possibility that relevant studies are missing from our meta-analysis. We consider the asymmetry to be low, with four data points from the article by Hurley *et al.* 1995^(66)^ standing out towards the lower left corner of the plot. Although the use of a funnel plot is not recommended when the statistical heterogeneity is large (> 50 %)^(79)^, as is the case for the present meta-analysis with I^2^ of 71 %, the low asymmetry in the funnel plot suggests that the risk for publication bias is low in the current meta-analysis.

### Dietary intake

If a complete or partial replacement of casein with proteins from fish affected the dietary intake, this may in turn have influenced biochemical parameters and physiological outcomes. Most of the articles reported either the energy intake or the amount of feed consumed (online Supplementary Table 1). The majority of studies, i.e. thirty-three of the thirty-nine articles included, reported no differences in dietary intake between the intervention and control groups^(31,32,34,36–44,47–49,51–54,56–67,69,70)^. The dietary intake was higher in Sprague Dawley rats fed a diet with 100 % of protein from carp muscles^(45)^ and was lower in Wistar rats consuming hydrolysed salmon by-products^(55)^ or hydrolysed muscles or by-products from sardine^(46)^ as the sole protein sources, when compared with rats fed casein diets. Two articles did not declare any information on the dietary intake^(35,68)^, and in one article the feed intake was measured but statistical testing was not performed^(50)^.

### Adiposity and bodyweight gain

A complete or partial replacement of casein with proteins from fish in the diet may affect adiposity in rodents, which in turn may affect the cholesterol metabolism. Adiposity was quantified in twenty-five articles (online Supplementary Table 1), and of these, twenty articles reported that the absolute or relative weights of adipose tissues were similar in rodents fed proteins from fish when compared with the control group^(31,32,36,37,41,42,45,48,49,52,54,56–62,65,69)^. A lower weight of one or multiple white adipose tissues was reported in Wistar rats consuming a diet with either hydrolysed salmon by-products as the sole protein source^(55)^, a diet with protamine as 25 % of protein^(70)^, or hydrolysed sardine by-products as 100 % of dietary protein^(46)^, and in C57BL/6J mice fed a diet with 20 % of protein from protamine^(53)^. One article described a higher relative weight of epididymal WAT in leptin-deficient *ob/ob* mice fed 100 % of protein from pollock muscles, but was similar to the control group with regard to relative weights of mesenteric, perirenal + retroperitoneal and inguinal WAT^(64)^.

Thirty-three articles provided information regarding bodyweight gain (often referred to as growth) during the intervention period. The reported bodyweight gain is presented in Supplementary Table 1, but is not further described here since differences in bodyweight do not necessarily reflect dissimilarities in adiposity or muscle mass between the experimental groups.

## Discussion

In the present systematic review, we collected information from thirty-nine relevant articles that investigated the effects of fish as a dietary protein source on cholesterol metabolism. The reviewed articles show a large variation in design in two of the components defined in our PICO strategy; i.e. a wide variety of rat and mouse strains and genetically modified rodents were used, and a wide variety of species, parts, preparations and doses of fish were explored in the interventions. The comparator was casein in an equivalent amount to that of the protein content in the intervention diets in all articles, and all included articles reported the effects of the intervention on the concentration of TC in serum or plasma. Based on the many differences in design used in the included articles, we expected that the reported results would not be uniform. To the best of our knowledge, this is the first systematic review and meta-analysis that explores the reported effects of protein from muscles and by-products from a variety of fish species on the circulating TC concentration.

The main meta-analysis, comprising thirty-nine articles and a total of 935 rodents, showed that intake of proteins from fish muscles or fish by-products resulted in a significantly lower circulating TC concentration when compared with their respective control group. Despite our effort to minimise the heterogeneity in design by applying a strict list of exclusion and inclusion criteria, the statistical heterogeneity was large, and we therefore conducted a series of subclass analyses. The subgroup meta-analyses showed no difference between the effects of consuming diets containing proteins from fish muscles or fish by-products, despite the larger sample size of the former (680 *v*. 255 rodents) and the large variety of by-products including fractions such as roe, milt, backbones and unspecified by-products. Also the subgroup comparison of the intervention groups consuming diets containing proteins from the *Gadidae, Salmonidae* and *Clupeidae* families showed that these were not significantly different. Likewise, a subgroup analysis of total replacement *v*. partial replacement (≤ 50 %) of casein with proteins from fish revealed no effect of high *v*. low intake of proteins from fish. An exciting finding from the subgroup meta-analyses was that the effect of dietary intake of protein from fish had the most marked effect in the subgroup consisting of genetically rodents which spontaneously develop hypertension after birth (i.e. the leptin-resistant Zucker *fa/fa* rats and the leptin-deficient *ob/ob* mice) and rodents fed diets enriched with cholesterol alone or in combination with cholate. Although none of the included studies investigated if serum/plasma TC was changed from baseline to endpoint of intervention, we expect the increase in TC over time to be most pronounced in this subgroup, and the subgroup meta-analysis showed that the effect of fish protein intervention was stronger in these rodents compared with the other rodents. This indicates that the potency for preventing an increase in TC was more pronounced than for lowering TC concentration. Although not directly transferable to humans, the finding that rodents with increased probability for developing hypercholesterolaemia had a circulating TC concentration that was in average 0·53 mmol/l (20 %) lower when consuming proteins from fish compared to controls should not be downplayed. According to Fager & Wiklund 1997^(80)^, a 3 % reduction in TC provides a 15 % reduction in CHD. Thus, the effect on circulating TC induced by fish and fish proteins may be clinically relevant and should be further explored in humans with a high risk for hypercholesterolaemia.

Several mechanisms of action have been suggested to explain how intake of fish or fish proteins can lead to a lower circulating TC concentration, including increased excretion of cholesterol and bile acids in faeces, lower endogenous cholesterol synthesis in the liver, increased uptake of LDL-cholesterol to the liver and reduced VLDL secretion from the liver. These pathways will be discussed below.

The possibility that fish proteins may lower the circulating TC concentration by reducing intestinal cholesterol absorption has been explored in several articles and comprises both the absorption after dietary intake and the reabsorption via the enterohepatic circulation. A decreased (re)absorption of cholesterol and bile acids will increase the faecal excretion of cholesterol and bile acids, thus reducing the cholesterol uptake to the liver and the hepatic secretion of cholesterol as VLDL, which will contribute to a lower TC concentration in the circulation. The daily faecal excretions of cholesterol and/or bile acids were measured in nineteen of the thirty-nine reviewed articles. A lower circulating TC concentration alongside higher faecal excretion of cholesterol and/or bile acids after fish protein intake was observed in rodents fed pollock muscles^(57–59,64)^, tuna muscles^(43)^, muscles or by-products from sardine^(46)^ or salmon protamine^(53,65,70)^. In line with this, it has been suggested that digestion products of pollock^(57–59,64,69)^, tuna^(43)^, sardine^(46)^ or salmon protamine^(53,65,70)^ may inhibit cholesterol and bile acid reabsorption, possibly by suppressing the micellar solubility of cholesterol^(58)^, thus resulting in more cholesterol and bile acids being excreted in faeces. In contrast, a lower circulating TC was not accompanied by changes in faecal excretion of cholesterol and/or bile acids after intake of cod muscles^(41)^, hydrolysed salmon muscles^(68)^ (presented in a separate publication^(81)^), baked salmon muscles^(31)^ or headed and gutted blue whiting protein powder^(44)^. Interestingly, these latter studies were all utilising obese Zucker *fa/fa* rats, which have mild cholestasis with reduced bile secretion in both basal and stimulated states^(72)^. Another possible mechanism of action is that fish proteins may increase the hepatic conversion of cholesterol to bile acids for faecal excretion, thus rendering less cholesterol available for secretion through stimulation of CYP7A1, which is the rate-determining enzyme for the conversion of cholesterol to bile acids. Two articles presented findings of lower serum TC combined with stimulated CYP7A1 and larger faecal bile acid excretion; both articles measured the CYP7A1 mRNA level in rodents fed pollock muscles^(64,69)^. Eight articles reported no change in hepatic CYP7A1 mRNA in rodents presenting lower circulating TC concentration after consumption of diets containing cod muscles^(41)^, pollock muscles^(57,59,67)^, hydrolysed salmon muscles^(68)^ (presented in a separate publication^(81)^), tuna muscles^(43)^ or protamine^(53,65)^. These findings indicate that with certain study designs, enhanced faecal excretion of cholesterol and/or bile acids may contribute to a lower circulating TC concentration in rodents consuming fish or fish proteins, whereas other mechanisms of action are more likely to be responsible for the lower serum concentrations in situations when faecal excretions of cholesterol and/or bile acids are not affected.

HMG-CoA reductase is the rate-determining enzyme for the hepatic synthesis of cholesterol, and a reduction in liver HMG-CoA reductase activity is the primary target for medical treatment of hypercholesterolaemia^(82)^ since this will reduce the amount of endogenous cholesterol available for secretion from the liver in VLDL particles. It is therefore of immense interest that two articles reported that a lower serum TC concentration corresponded with lower hepatic mRNA expression of HMG-CoA reductase after cod muscle consumption^(41)^ and lower HMG-CoA reductase concentration in the liver after consumption of blue whiting^(44)^. Both experiments were conducted in obese Zucker *fa/fa* rats and indicated a lower endogenous production of cholesterol. Mice fed protamine had a higher HMG-CoA reductase gene expression in the liver and a lower serum TC concentration^(53)^, and Zucker *fa/fa* rats fed salmon muscle proteins had a higher hepatic HMG-CoA reductase gene activity combined with a lower plasma TC concentration^(68)^. Others found no difference in the HMG-CoA reductase mRNA level between rodents with lower circulating TC concentration consuming proteins from fish and their corresponding control groups^(43,57,59,64,69)^. Thus, a lower HMG-CoA reductase activity may explain the lower serum TC concentration in only a few of the reviewed articles.

An up-regulation of the LDL-receptor, especially in the liver, will increase the removal of LDL-cholesterol and make cholesterol available for faecal excretion or storage in the liver; however, a high hepatic TC concentration may down-regulate the LDL-receptor transcription^(83)^. A lower serum TC concentration combined with up-regulation of the LDL-receptor gene expression in the liver was seen only in two articles, i.e. in Sprague Dawley rats fed pollock muscles without affecting LDL-cholesterol concentration^(67)^, and in C57BL/6J mice (LDL-cholesterol was not assessed)^(53)^. Others report that lower circulating concentrations of TC^(57,59,64,68,69,81)^ or LDL-cholesterol^(57,59,65,69)^ were observed with no difference in hepatic LDL-receptor mRNA level when compared with their respective control groups. A down-regulated LDL-receptor gene expression^(41)^ and protein concentration^(44)^ were observed in livers from Zucker *fa/fa* rats fed cod or blue whiting, respectively, possibly as a consequence of the lower LDL-cholesterol and TC concentrations in serum. The lack of effect on plasma TC concentration after feeding diets containing salmon proteins in the hypercholesterolemic strains LDLR^−/−^/ApoB^100/100^ mice^(37)^ and apoE^–/–^ mice^(35)^, which have reduced clearance of LDL, also indicates that the fish proteins’ modifying impact on circulating TC concentration may be via the LDL receptor, which binds to both apoB100 and apoE. Thus, the role of the LDL receptor in the regulation of circulating TC and LDL-cholesterol after fish protein intake is difficult to assess.

Cholesterol that is endogenously produced in the liver or taken up via the LDL receptor to the liver, may have many fates, including being stored in the liver as cholesteryl esters, being removed from the liver in bile as cholesterol or after being transformed to bile acids, or being secreted as VLDL (the triacylglycerol-rich precursor for LDL). The esterification of cholesterol is catalysed by the rate-determining enzyme SOAT2. A lower circulating TC concentration^(43,59,67,69)^ or an elevated hepatic TC content^(67)^ were observed concurrently with no difference in SOAT2 mRNA level when compared with controls. However, a lower hepatic gene expression of SOAT2 may reflect lower uptake of cholesterol via the LDL receptor and lower endogenous cholesterol synthesis, resulting in lower VLDL secretion and thus a lower serum cholesterol concentration, as suggested for Zucker *fa/fa* rats fed cod muscles where lower hepatic gene expressions of both LDL receptor and HMG-CoA reductase were observed^(41)^. This is supported by the finding of lower VLDL secretion in Sprague Dawley rats fed cod muscle proteins^(40)^. Others found lower SOAT2 activity in the liver of Zucker *fa/fa* rats fed salmon muscles and suggested that the observation of lower plasma TC concentration was caused by lower VLDL secretion^(68)^. Thus, SOAT2 may be involved in regulating circulating TC concentration, at least in Zucker *fa/fa* rats.

It has been suggested that the amino acid composition of fish proteins can be an explanation for a beneficial effect on cholesterol metabolism, as the low ratios of methionine/glycine and lysine/arginine in soya, potatoes and rice proteins are believed to contribute to their cholesterol-lowering effect^(84,85)^. Lower methionine/glycine^(42,46,68)^ and lysine/arginine ratios^(42,43,46,68)^ were also observed in fish proteins when compared with casein, concomitant with a lower circulating TC concentration, and the authors of these articles suggested that the hypocholesterolemic effect of fish proteins may be mediated through these differences in contents of amino acids. In other articles, especially in those using partial replacement of dietary casein, little or no differences in amino acids were seen between the fish protein diets and the control diets, and some authors doubt that there is any connection between these amino acid ratios and the cholesterol-lowering effect of fish proteins^(31,41,42,57,59)^.

A wide range of bioactive peptides has been identified in fish muscles and by-products^(86,87)^, and among the reviewed articles, several hypothesise that bioactive peptides in fish proteins are responsible for the observed effects on cholesterol metabolism^(31,35–37,42,44,46,47,50,52,54,56)^. Still, the presence of bioactive peptides (as motifs) in fish was investigated only in two articles^(42,44)^. Of particular interest in the current setting is the two hypocholesterolemic peptides Gly-Gly-Val, which is a strong inhibitor of HMG-CoA reductase activity *in vitro* ^(88)^, and Ile-Ile-Ala-Glu-Lys, which is a strong stimulator of CYP7A1^(89)^. The presence of Gly-Gly-Val in blue whiting may have contributed to the lower hepatic HMG-CoA reductase concentration and, thereby, to the lower cholesterol concentrations in the liver and consequently in the serum of rats fed a diet containing blue whiting^(44)^. Ile-Ile-Ala-Glu-Lys has a greater cholesterol-lowering capacity than *β*-sitosterol in rats^(90)^, and the lower LDL-cholesterol concentration in rats fed herring hydrolysate may be due to the presence of Ile-Ile-Ala-Glu-Lys, thus stimulating the conversion of cholesterol to bile acids and making less cholesterol available for VLDL secretion, and, in turn, a lower concentration of LDL-cholesterol^(42)^.

A change in adiposity has a strong influence on serum TC concentration. The endogenous cholesterol synthesis is linearly correlated with body fat stores^(91)^, and a loss of excess body weight will efficiently lower circulating TC in humans with overweight or obesity^(92)^. Of the five articles reporting differences in adiposity between the fish protein intervention group and the corresponding control group, a lower adiposity was associated with lower serum TC concentration in three studies; in Wistar rats fed hydrolysed sardine by-products as the sole protein source^(46)^ or 25 % of dietary protein as protamine^(70)^, and in C57BL/6J mice fed a diet with 20 % of protein from protamine^(53)^. In the only article that reported a higher relative weight of epididymal WAT (but similar relative weights of mesenteric, perirenal + retroperitoneal and inguinal WAT to those of the control group), i.e. in leptin-deficient *ob/ob* mice fed 100 % of protein from pollock muscles, the serum TC concentration was actually lower compared with the corresponding control mice^(64)^. Twenty articles reported that the adiposity was similar between the intervention group(s) and the corresponding control group, and the circulating TC concentration was lower for half of these when compared with controls^(31,32,41,45,52,57–59,65,69)^. Thus, it seems that differences in adiposity may not be a strong determinant for the circulating TC concentration after fish protein feeding in the reviewed articles.

High-fat diets and/or high-carbohydrate diets and diets with added cholesterol alone or in combination with cholate mimic high energy Western obesogenic and cholesterologenic diets and are relevant when fed to rodents as models of high cholesterol in humans^(93,94)^. Obese rodents such as the obese Zucker *fa/fa* rat which has a mutated leptin receptor^(95)^ and the leptin-deficient *ob/ob* mouse^(76)^, are also relevant models for human hypercholesterolaemia. Likewise, the apolipoprotein E null mouse^(73)^ and the LDLR-deficient mouse^(74)^ are relevant models of hypercholesterolaemia since LDL removal is inhibited and thus the cholesterol metabolism is disturbed. The cholesterol metabolism in rodents is in many aspects similar to that in humans, as the cholesterol concentrations in circulation, liver and extrahepatic tissues are controlled through pathways including the activities of HMG-CoA reductase and LDL-receptor, through the faecal excretion of cholesterol and bile acids, and is strongly influenced by the dietary intake of cholesterol and cholate. For both rodents and humans, the liver is the primary site for endogenous cholesterol production, and faecal excretion of bile acids is the major route of cholesterol removal from the body. Still, there are substantial differences between rodents and humans that limit the reliability and translation of findings from rodents to humans. Most importantly, mice and rats are naturally deficient in cholesteryl ester transfer protein. Whereas rodents use HDL as the major cholesterol transporter^(97)^, humans transport the majority of cholesterol in LDL particles^(96)^. Although dietary studies in rodents are relevant for humans, the findings in rodents must be interpreted with caution regarding the relevance to human physiology.

The interpretations of the findings in the current systematic review and meta-analysis may be impacted by a variety of factors concerning the design of the reviewed studies, including the gender of the animals. Male mice develop obesity faster and to a larger degree than female mice in response to an obesogenic diet, whereas obesity is more prevalent in women^(98)^. In rats, the gender difference is generally less pronounced in response to diet-induced obesity^(98)^, although female obese Zucker *fa/fa* rats have higher hepatic HMG-CoA reductase activity compared with male littermates^(99)^. Only two of the included articles reported the use of female rodents; both were studies in mice^(34,35)^, and the predominance of studies conducted in male rodents make the results from the present study less generalisable to humans. The interpretation of the included findings is restricted by the limited number of articles, combined with the large variety in rodent models, fish species, the use of muscles or by-products, the dose of fish protein and the duration of the interventions. Although several articles proposed possible mechanisms of action behind a lower circulating TC concentration after consuming fish proteins, many articles did not provide a mechanistic explanation for their findings or did not provide information relevant to explaining any changes in the cholesterol metabolism.

It is difficult to assess the risk of bias for the included studies since most of the entries in the SYRCLE’s risk of bias tool^(26)^ were not addressed in the articles and were graded as having an unclear risk of bias to the reported findings. Still, we would like to especially comment on the lack of information regarding blinding in relation to performance bias (#5) and detection bias (#7) in all but one of the included articles^(41)^. The lack of blinding may involuntarily affect the researchers’ evaluation of the study outcomes and adversely affect published research. Others also report of lack of blinding in experimental studies, and found that non-blinded studies have more statistically significant findings^(100)^. However, we consider the included studies to be of high quality with regard to the primary aim of the present systematic review and meta-analysis (i.e. effects of proteins from fish on circulating TC concentration), based on the selected items from the CAMARADES checklist^(28)^ and the ARRIVE 2.0 guidelines^(29)^.

### Conclusion

The meta-analysis provided evidence that a dietary intake of proteins from fish muscles or fish by-products results in lower circulating TC concentration in rodents. We consider the publication bias to be low for the meta-analysis, and the quality of the included studies to be high but with an uncertain risk of bias. The systematic review indicates that a beneficial effect on cholesterol metabolism may be regulated through at least two distinct metabolic pathways depending on the diet composition and rat model rather than the type or fraction of fish proteins consumed. In normocholesterolemic rodents fed diets with fish proteins, the mechanism of action was suggested to be regulated through increased faecal removal of TC and/or bile acids in some of the articles. In the genetically hypercholesterolemic Zucker *fa/fa* rats, the reported findings suggest that the lower circulating TC concentration after consumption of proteins from fish may be mediated through down-regulation of the activity of hepatic HMG-CoA reductase and thus down-regulation of the endogenous cholesterol synthesis.

Proteins from fish muscles and by-products show promise as a functional ingredient by promoting a lower circulating TC concentration, thus reducing one of the most important risk factors for developing CVD. Fish proteins as part of the diet or taken as a dietary supplement may be relevant for preventing hypercholesterolaemia in humans with increased risk caused by genetic dispositions or a cholesterologenic diet.

## Supplementary material

For supplementary material accompanying this paper visit https://doi.org/10.1017/S000711452200349X. click here to view supplementary material
