# Supplementary material for: Effects of diets containing proteins from fish muscles or fish by-products on the circulating cholesterol concentration in rodents: a systematic review and meta-analysis
Source: Br J Nutr. 2022 Oct 21;130(3):389–410. doi: 10.1017/S000711452200349X (PMC10331438; doi:10.1017/S000711452200349X)
Supplement: Supplementary file 1 [file S000711452200349Xsup.zip › S000711452200349Xsup001.docx]

**Supplementary Table 1:** Study characteristics (type and fraction of fish used, length of intervention period, diet availability, prandial state at euthanasia) and outcomes (dietary intake, bodyweight gain, adiposity)

| Ref. | **Fish species (w. Latin name when provided) or genus, part of fish used and processing** | **Duration of intervention period** | **Diet availability** | **Prandial state at euthanasia** | **Dietary intake in fish protein group(s) compared to casein group** | **Bodyweight gain during the intervention period in fish protein group(s) compared to the casein group** | **Adiposity in fish protein group(s) compared to casein group** |
| --- | --- | --- | --- | --- | --- | --- | --- |
| ^(^[^39^](#_ENREF_39)^)^ | Cod muscles were lyophilised and defatted | 3 weeks | Ad libitium | Fasted 6-8 hrs | Feed intake: NS | NS in cholesterol-free cod diet, weight gain was larger in rats fed a cholesterol-enriched diet containing cod | N/A |
| ^(^[^63^](#_ENREF_63)^)^ | Cod muscles were lyophilised, defatted and powdered | 3 weeks | Ad libitum | Non-fasted | Feed intake: NS | NS | N/A |
| ^(^[^66^](#_ENREF_66)^)^ | Cod muscles were defatted and lyophilised | 28 days | Ad libitium | n 10 fasted (16 hrs) and n 10 non-fasted rats in each dietary group | Feed intake: NS | NS | N/A |
| ^(^[^40^](#_ENREF_40)^)^ | Cod muscles were defatted and lyophilised | 28 days | Ad libitium | Fasted 12 hrs | Feed intake: NS | NS | N/A |
| ^(^[^68^](#_ENREF_68)^)^ | Both experiments:  Atlantic salmon muscles were hydrolysed | Exp 1:  11–12 d  Exp 2:  22- 23 d. | Both experiments:  Ad libitium | Both experiments:  Fasted: N/A | Both experiments:  N/A | Both experiments:  N/A | Both experiments:  N/A |
| ^(^[^67^](#_ENREF_67)^)^ | Both experiments:  Alaska pollock (Theragra chalcogramma) muscles were defatted and lyophilised | Exp 1: 20 days  Exp 2: 22 days | Exp. 1:  Feed intake was slightly restricted  Exp. 2:  Feed intake was slightly restricted | Exp. 1:  Fasted overnight (12 hrs)  Exp. 2:  Non-fasted | Both experiments:  Feed intake: NS | Exp. 1:  NS  Exp. 2:  Higher in the Alaska pollock protein group | Both experiments:  N/A |
| ^(^[^36^](#_ENREF_36)^)^ | Herring roe was defatted and dried | Pre-diet: 19d with 17% lard.  Intervention: 3 weeks | Ad libitium | Fasted overnight | Feed intake: NS | NS | Abdominal fat pad (epidermal adipose tissues) relative weight: NS |
| ^(^[^69^](#_ENREF_69)^)^ | Alaska pollock (Theragra chalcogramma) muscles were defatted and dried | 4 weeks | N/A | N/A | Energy intake: NS | NS | Abdominal relative WAT weight: NS |
| ^(^[^49^](#_ENREF_49)^)^ | Saithe (Pollachius virens, L.) frames were hydrolysed | 26 days | Pair-feeding (no further information provided) | Fasted 24 hrs | Energy intake: NS | Lower in the saithe group | Sum of perirenal + retroperitoneal adipose tissue weights: NS |
| ^(^[^43^](#_ENREF_43)^)^ | Tuna muscles were defatted and dried | 3 weeks | Ad libitium | Fasted overnight | Feed intake: NS | N/A | N/A |
| ^(^[^38^](#_ENREF_38)^)^ | Cod muscles were lyophilised and defatted | 28 days | Ad libitium | Fasted 12 hrs | Energy intake: NS  Feed intake: NS | NS | N/A |
| ^(^[^61^](#_ENREF_61)^)^ | Alaska pollock (Theragra chalcogramma) muscles were defatted and lyophilised | 4 weeks | N/A | Non-fasted | Feed intake: NS | NS | Perirenal, epididymal, and mesenteric relative WAT weights: NS  Total body fat, %: NS |
| ^(^[^65^](#_ENREF_65)^)^ | Salmon protamine | 4 weeks | Ad libitum | Non-fasted | Feed intake: NS  Energy intake: NS | NS | The relative weight of epididymal, mesenteric and perirenal + retroperitoneal: NS |
| ^(^[^58^](#_ENREF_58)^)^ | Alaska pollock (Theragra chalcogramma) muscles were defatted and lyophilised | 4 weeks | N/A | Non-fasted | Feed intake: NS | NS | Epididymal relative WAT weight: NS |
| ^(^[^55^](#_ENREF_55)^)^ | Both experiments:  Atlantic salmon (Salmo salar, L.) by-products were hydrolysed | Exp 1:  46 days  Exp 2:  25 days | Exp 1:  Ad libitum  Exp 2:  Pair-feeding (no further information provided) | Exp 1:  Non-fasted  Exp 2:  Non-fasted | Exp 1  Energy intake: lower in the salmon group  Exp2:  N/A | Exp 1:  NS  Exp 2:  N/A | Exp 1: Epididymal and mesenteric WAT weights: lower in the salmon group  Exp 2: N/A |
| ^(^[^51^](#_ENREF_51)^)^ | Sardine (Sardina pilchardus) muscles were processed into a presscake, and protein from the presswater was incorporated into the presscake-meal. The final product was defatted | Pre-diet: 10d with 1.5% of cholesterol and 0.75% cholic acid.  Intervention period: 4 weeks | N/A | Fasted overnight | Feed intake: NS | N/A | N/A |
| ^(^[^34^](#_ENREF_34)^)^ | Herring (Clupea harengus) roe and milt were spray-dried | 2 weeks | Ad libitum | Fasted (duration not stated) | Feed intake: NS | Higher in both the herring roe and the herring milt groups | N/A |
| ^(^[^59^](#_ENREF_59)^)^ | Alaska pollock (Theragra chalcogramma) muscles were defatted, hydrolysed and dried | 4 weeks | Ad libitum | Non-fasted | Energy intake: NS | NS | Sum of epididymal + mesenteric + perinephric relative WAT weights: NS |
| ^(^[^57^](#_ENREF_57)^)^ | Alaska pollock (Theragra chalcogramma) muscles were defatted and dried | 4 weeks | N/A | Non-fasted | Feed intake: NS | NS | Epididymal, mesenteric, and perirenal + retroperitoneal relative WAT weights: NS |
| ^(^[^35^](#_ENREF_35)^)^ | Salmon by-products (spines) were hydrolysed | 12 weeks | N/A | N/A  Blood was collected after an overnight fast one week before euthanasia for plasma TC analysis | N/A | NS | N/A |
| ^(^[^62^](#_ENREF_62)^)^ | Alaska pollock (Theragra chalcogramma) muscles were defatted and lyophilised | 8 weeks | Ad libitum | Non-fasted | Energy intake: NS | NS | Perirenal, epididymal, and mesenteric relative WAT weights: NS  Total body fat, %: NS |
| ^(^[^60^](#_ENREF_60)^)^ | Alaska pollock (Theragra chalcogramma) muscles were defatted and lyophilised | 4 weeks | N/A | Non-fasted | Feed intake: NS | NS | Epididymal and perirenal + retroperitoneal relative WAT weights; NS |
| ^(^[^50^](#_ENREF_50)^)^ | E1: salmon spine (hydrolysed, Acid Protease A)  E2: salmon spine (hydrolysed, Umamizyme)  E4: Salmon backbone and heads (hydrolysed, Alcalase) | 6 weeks | Ad libitum | Fasted overnight | Feed intake: not statistically tested | Lower in the E1 and E4 groups, but E2 was similar to casein | N/A |
| ^(^[^70^](#_ENREF_70)^)^ | Salmon protamine | 4 weeks | N/A | N/A | Energy intake: NS | NS | The relative weight of epididymal WAT was lower in the protamine group. NS for mesenteric, perirenal+ retroperitoneal, and total WAT weight |
| ^(^[^37^](#_ENREF_37)^)^ | Atlantic salmon (Salmo salar) muscles were hydrolysed | 12 weeks | Ad libitium | Fasted 6 hrs | Energy intake: NS | N/A | Epididymal, inguinal and retroperitoneal WAT weights: NS |
| ^(^[^45^](#_ENREF_45)^)^ | Carp dorsal muscles were defatted and lyophilised | 1 week | Ad libitium  Fasted 4 hrs |  | Feed intake: higher in the carp group | NS | Epididymal WAT weight and relative weight: NS |
| ^(^[^42^](#_ENREF_42)^)^ | Atlantic salmon (Salmo salar) backbones and Atlantic herring (Clupea harengus) by-products (heads, guts, backbones) were hydrolysed and dried | 4 weeks | Ad libitium | Fasted 12 hrs | Energy intake: NS | Higher in the salmon group, the herring group was similar to the casein group | Epididymal WAT relative weight: NS |
| ^(^[^54^](#_ENREF_54)^)^ | Both experiments:  Alaska pollock (Theragra chalcogramma) muscles and tuna (Thunnus orientalis) muscles were defatted and lyophilised | Both experiments:  28 days | Both experiments:  Ad libitium | Both experiments:  Non-fasted | Both experiments:  Feed intake: NS | Both experiments:  NS | Both experiments: Perirenal, mesenteric and epididymal relative WAT^1^ weights: NS |
| ^(^[^31^](#_ENREF_31)^)^ | Atlantic salmon (Salmo salar) muscles (raw or baked), were lyophilised | 4 weeks | Ad libitium | Fasted overnight (12 hrs) | Energy intake: NS | NS | Epididymal relative WAT weight: NS |
| ^(^[^56^](#_ENREF_56)^)^ | Tuna (Thunnus orientalis) dark muscles were defatted and lyophilised | 28 days | Ad libitium | Non-fasted | Feed intake: NS | NS | Inguinal, mesenteric, epididymal, and sum of perirenal + retroperitoneal relative WAT weights: NS |
| ^(^[^44^](#_ENREF_44)^)^ | Blue whiting (Micromesistius poutassou) was headed and gutted, and the water-soluble fraction (press liquid) was lyophilised | 5 weeks | Ad libitium | Fasted overnight (12 hrs) | Energy intake: NS | NS | N/A |
| ^(^[^41^](#_ENREF_41)^)^ | Atlantic cod (Gadus morhua) muscles were baked and lyophilised | 4 weeks | Ad libitium | Fasted 12 hrs | Energy intake: NS | NS | Sum of epididymal, renal and retroperitoneal relative WAT weights: NS |
| ^(^[^32^](#_ENREF_32)^)^ | Atlantic salmon (Salmo salar) muscles were baked and lyophilised | Both experiments:  4 weeks | Both experiments:  Ad libitium | Both experiments:  Fasted 12 hrs | Both experiments:  Energy intake: NS | Both experiments:  N/A | Both experiments:  Sum of epididymal, renal and retroperitoneal relative WAT weights: NS |
| ^(^[^46^](#_ENREF_46)^)^ | Sardine (Sardina pilchardus) presscake meals from muscles and by-products (viscera, heads, skins and edges) were defatted, hydrolysed and lyophilised | Pre-diet: 3 months with 20% mutton fat. Intervention period: 4 weeks | Ad libitium | Fasted overnight | Feed intake: lower in the sardine fillet protein group and in the sardine by-product group | Lower in both sardine hydrolysate groups | The adipose tissue (not specified) relative weight: lower in the sardine by-products hydrolysate group, NS for the sardine fillet hydrolysate group |
| ^(^[^47^](#_ENREF_47)^)^ | Herring milt was hydrolysed and lyophilised | Pre-diet: 6 weeks with 32% lard.  Intervention period: 10 weeks | Ad libitum feeding/controlled intake: N/A | Fasted overnight | Feed intake: NS | N/A | N/A |
| ^(^[^52^](#_ENREF_52)^)^ | Herring milt dry powder (blended, cooked over low heat and dried)  Herring milt protein hydrolysate (hydrolysed and lyophilised) | Pre-diet: 7 weeks with 32% lard  Intervention period: 9 weeks | N/A | Fasted overnight | Feed intake: NS | Lower in both herring groups | Visceral fat/body weight: NS |
| ^(^[^64^](#_ENREF_64)^)^ | Alaska pollock (Theragra  chalcogramma) muscles were lyophilised, defatted, air-dried and ground | 6 weeks | Pair-feeding (no further information provided) | Non-fasted | Feed intake: NS | Lower in the Alaska pollock group | The relative weight of epididymal WAT was higher in the Alaska pollock group. Mesenteric, perirenal+  retroperitoneal, and inguinal WAT: NS |
| ^(^[^48^](#_ENREF_48)^)^ | Alaska pollock (Theragra chalcogramma) muscles were defatted and air-dried | 8 weeks | Ad libitium | Fasted overnight | Feed intake: NS | NS | Epididymal, mesenteric, perirenal and inguinal relative WAT weights: NS |
| ^(^[^53^](#_ENREF_53)^)^ | Salmon protamine | 50 days | Ad libitum | Fasted 22 hrs | Feed intake: NS | Lower in the protamine group | The relative weights of subcutaneous, perirenal and total WAT: lower in the protamine group. NS for epididymal and mesenteric WAT |

NS; not statistically significant, N/A, data not available, WAT, white adipose tissue
