# Supplementary material for: Effects of diets containing proteins from fish muscles or fish by-products on the circulating cholesterol concentration in rodents: a systematic review and meta-analysis
Source: Br J Nutr. 2022 Oct 21;130(3):389–410. doi: 10.1017/S000711452200349X (PMC10331438; doi:10.1017/S000711452200349X)
Supplement: Supplementary file 1 [file S000711452200349Xsup.zip › S000711452200349Xsup002.docx]

**Supplementary Table 2:** Evaluation of the risk of bias for the included articles (Yes; low risk of bias, No; high risk of bias, Unclear; Unclear risk of bias)

|  | SYRCLES items with signalling questions | | | | | | | | | |
| --- | --- | --- | --- | --- | --- | --- | --- | --- | --- | --- |
|  | 1. Sequence generation (selection bias) | 2. Baseline characteristics (selection bias) | 3. Allocation concealment (selection bias) | 4. Random housing (performance bias) | 5. Blinding (performance bias) | 6. Random outcome assessment (detection bias) | 7. Blinding (detection bias) | 8. Incomplete outcome data (attrition bias) | 9. Selective outcome reporting (reporting bias) | 10. Other sources of bias |
|  | Was the allocation sequence adequately generated and applied? | Were the groups similar at baseline, or were they adjusted for confounders in the analysis? | Was the allocation adequately concealed? | Were the animals randomly housed during the experiment? | Were the caregivers and/or investigators blinded from knowledge which intervention each animal received during the experiment? | Were animals selected at random for outcome assessment? | Was the outcome assessor blinded? | Were incomplete outcome data adequately addressed? | Are reports of the study free of selective outcome reporting? | Was the study apparently free of other problems that could result in a high risk of bias? |
| ^(^[^39^](#_ENREF_39)^)^ | No | Unclear | Unclear | Yes | Unclear | Unclear | Unclear | No | Unclear | Unclear |
| ^(^[^63^](#_ENREF_63)^)^ | No | Unclear | Unclear | Yes | Unclear | Unclear | Unclear | No | Unclear | Unclear |
| ^(^[^66^](#_ENREF_66)^)^ | No | Unclear | Unclear | Yes | Unclear | Unclear | Unclear | No | Unclear | Unclear |
| ^(^[^40^](#_ENREF_40)^)^ | No | Unclear | Unclear | Yes | Unclear | Unclear | Unclear | Unclear | Unclear | Unclear |
| ^(^[^68^](#_ENREF_68)^)^ | No | Unclear | Unclear | Yes | Unclear | Unclear | Unclear | Yes | Unclear | Unclear |
| ^(^[^67^](#_ENREF_67)^)^ | No | Unclear | Unclear | Yes | Unclear | Unclear | Unclear | Yes | Unclear | Unclear |
| ^(^[^36^](#_ENREF_36)^)^ | No | Yes | Unclear | Yes | Unclear | Unclear | Unclear | Yes | Unclear | Unclear |
| ^(^[^69^](#_ENREF_69)^)^ | No | Yes | Unclear | Yes | Unclear | Unclear | Unclear | Unclear | Unclear | Unclear |
| ^(^[^49^](#_ENREF_49)^)^ | No | Yes | Unclear | Yes | Unclear | Unclear | Unclear | No | Unclear | Unclear |
| ^(^[^43^](#_ENREF_43)^)^ | No | Yes | Unclear | Yes | Unclear | Unclear | Unclear | Yes | Unclear | Unclear |
| ^(^[^38^](#_ENREF_38)^)^ | No | Yes | Unclear | Yes | Unclear | Unclear | Unclear | No | Unclear | Unclear |
| ^(^[^61^](#_ENREF_61)^)^ | No | Yes | Unclear | Yes | Unclear | Unclear | Unclear | Yes | Unclear | Unclear |
| ^(^[^65^](#_ENREF_65)^)^ | No | Yes | Unclear | Yes | Unclear | Unclear | Unclear | Yes | Unclear | Unclear |
| ^(^[^58^](#_ENREF_58)^)^ | No | Yes | Unclear | Yes | Unclear | Unclear | Unclear | Yes | Unclear | Unclear |
| ^(^[^55^](#_ENREF_55)^)^ | No | Unclear | Unclear | Yes | Unclear | Unclear | Unclear | Unclear | Unclear | Unclear |
| ^(^[^51^](#_ENREF_51)^)^ | No | Unclear | Unclear | Yes | Unclear | Unclear | Unclear | Yes | Unclear | Unclear |
| ^(^[^34^](#_ENREF_34)^)^ | No | Yes | Unclear | Yes | Unclear | Unclear | Unclear | Yes | Unclear | Unclear |
| ^(^[^59^](#_ENREF_59)^)^ | No | Yes | Unclear | Yes | Unclear | Unclear | Unclear | Yes | Unclear | Unclear |
| ^(^[^57^](#_ENREF_57)^)^ | No | Yes | Unclear | Yes | Unclear | Unclear | Unclear | Yes | Unclear | Unclear |
| ^(^[^35^](#_ENREF_35)^)^ | No | Yes | Unclear | Yes | Unclear | Unclear | Unclear | No | Unclear | Unclear |
| ^(^[^62^](#_ENREF_62)^)^ | No | Yes | Unclear | Yes | Unclear | Unclear | Unclear | Yes | Unclear | Unclear |
| ^(^[^60^](#_ENREF_60)^)^ | No | Yes | Unclear | Yes | Unclear | Unclear | Unclear | Yes | Unclear | Unclear |
| ^(^[^50^](#_ENREF_50)^)^ | No | Yes | Unclear | Yes | Unclear | Unclear | Unclear | No | Unclear | Unclear |
| ^(^[^70^](#_ENREF_70)^)^ | No | Yes | Unclear | Yes | Unclear | Unclear | Unclear | Yes | Unclear | Unclear |
| ^(^[^37^](#_ENREF_37)^)^ | No | Yes | Unclear | Yes | Unclear | Unclear | Unclear | No | Unclear | Unclear |
| ^(^[^45^](#_ENREF_45)^)^ | No | Yes | Unclear | Yes | Unclear | Unclear | Unclear | Yes | Unclear | Unclear |
| ^(^[^42^](#_ENREF_42)^)^ | No | Yes | Unclear | Yes | Unclear | Unclear | Unclear | Yes | Unclear | Unclear |
| ^(^[^54^](#_ENREF_54)^)^ | No | Yes | Unclear | Yes | Unclear | Unclear | Unclear | Yes | Unclear | Unclear |
| ^(^[^31^](#_ENREF_31)^)^ | No | Yes | Unclear | Yes | Unclear | Unclear | Unclear | Yes | Unclear | Unclear |
| ^(^[^56^](#_ENREF_56)^)^ | No | Yes | Unclear | Yes | Unclear | Unclear | Unclear | Yes | Unclear | Unclear |
| ^(^[^44^](#_ENREF_44)^)^ | No | No | Unclear | Yes | Unclear | Unclear | Unclear | Yes | Unclear | Unclear |
| ^(^[^41^](#_ENREF_41)^)^ | No | Yes | Unclear | Yes | Yes | Unclear | Yes | Yes | Unclear | Unclear |
| ^(^[^32^](#_ENREF_32)^)^ | No | Yes | Unclear | Yes | Unclear | Unclear | Unclear | Yes | Unclear | Unclear |
| ^(^[^46^](#_ENREF_46)^)^ | No | Yes | Unclear | Yes | Unclear | Unclear | Unclear | Yes | Unclear | Unclear |
| ^(^[^47^](#_ENREF_47)^)^ | No | Yes | Unclear | Yes | Unclear | Unclear | Unclear | No | Unclear | Unclear |
| ^(^[^52^](#_ENREF_52)^)^ | No | Yes | Unclear | Yes | Unclear | Unclear | Unclear | No | Unclear | Unclear |
| ^(^[^64^](#_ENREF_64)^)^ | No | Yes | Unclear | Yes | Unclear | Unclear | Unclear | Yes | Unclear | Unclear |
| ^(^[^48^](#_ENREF_48)^)^ | No | Yes | Unclear | Yes | Unclear | Unclear | Unclear | Yes | Unclear | Unclear |
| ^(^[^53^](#_ENREF_53)^)^ | No | Yes | Unclear | Yes | Unclear | Unclear | Unclear | Yes | Unclear | Unclear |
