# Supplementary material for: Effects of diets containing proteins from fish muscles or fish by-products on the circulating cholesterol concentration in rodents: a systematic review and meta-analysis
Source: Br J Nutr. 2022 Oct 21;130(3):389–410. doi: 10.1017/S000711452200349X (PMC10331438; doi:10.1017/S000711452200349X)
Supplement: Supplementary file 1 [file S000711452200349Xsup.zip › S000711452200349Xsup003.docx]

**Supplementary Table 3:** Study quality checklist for the included studies. Studies were scored with one point per item when the information was provided, and with zero points if the information was missing.

| Refs. | 1.  Peer reviewed publication | 2.  Animal model (specie, strain) | 3.  Sex of the experimental animals | 4. Husbandry conditions and actions to improve animal welfare of the experimental animals (e.g., environmental enrichment) | 5. Description of the procedures, including when it was done, any preparation of the animals (prandial status, use of anaesthesia) | 6. Description of analysis, including name and brand of assays and kits | 7.  Details of the statistical methods used for each analysis | 8. Summary/descriptive statistics for each experimental group, with a measure of variability where applicable | 9. Compliance with animal welfare regulations | 10. Statement of potential conflict of interests | Total score (of 10) |
| --- | --- | --- | --- | --- | --- | --- | --- | --- | --- | --- | --- |
| ^(^[^39^](#_ENREF_39)^)^ | 1 | 1 | 1 | 0 | 1 | 1 | 1 | 1 | 0 | 0 | 7 |
| ^(^[^63^](#_ENREF_63)^)^ | 1 | 1 | 1 | 0 | 1 | 1 | 1 | 1 | 0 | 0 | 7 |
| ^(^[^66^](#_ENREF_66)^)^ | 1 | 1 | 1 | 0 | 1 | 1 | 1 | 1 | 1 | 0 | 8 |
| ^(^[^40^](#_ENREF_40)^)^ | 1 | 1 | 1 | 0 | 1 | 1 | 1 | 1 | 1 | 0 | 8 |
| ^(^[^68^](#_ENREF_68)^)^ | 1 | 1 | 1 | 0 | 0.5^a^ | 1 | 1 | 1 | 1 | 0 | 7.5 |
| ^(^[^67^](#_ENREF_67)^)^ | 1 | 1 | 1 | 0 | 1 | 1 | 1 | 1 | 1 | 0 | 8 |
| ^(^[^36^](#_ENREF_36)^)^ | 1 | 1 | 0 | 0 | 1 | 0 | 1 | 1 | 1 | 0 | 6 |
| ^(^[^69^](#_ENREF_69)^)^ | 1 | 1 | 1 | 0 | 0.5 ^a^ | 0 | 1 | 1 | 1 | 0 | 6.5 |
| ^(^[^49^](#_ENREF_49)^)^ | 1 | 1 | 1 | 0 | 1 | 0 | 1 | 1 | 1 | 0 | 7 |
| ^(^[^43^](#_ENREF_43)^)^ | 1 | 1 | 1 | 0 | 1 | 0 | 1 | 1 | 1 | 0 | 7 |
| ^(^[^38^](#_ENREF_38)^)^ | 1 | 1 | 1 | 0 | 1 | 1 | 1 | 1 | 1 | 1 | 9 |
| ^(^[^61^](#_ENREF_61)^)^ | 1 | 1 | 1 | 0 | 1 | 1 | 1 | 1 | 1 | 0 | 8 |
| ^(^[^65^](#_ENREF_65)^)^ | 1 | 1 | 1 | 0 | 1 | 0 | 1 | 1 | 1 | 0 | 7 |
| ^(^[^58^](#_ENREF_58)^)^ | 1 | 1 | 1 | 0 | 1 | 0 | 1 | 1 | 1 | 0 | 7 |
| ^(^[^55^](#_ENREF_55)^)^ | 1 | 1 | 1 | 0 | 0.5^b^ | 0 | 1 | 1 | 1 | 0 | 6.5 |
| ^(^[^51^](#_ENREF_51)^)^ | 1 | 1 | 1 | 0 | 1 | 1 | 1 | 1 | 1 | 0 | 8 |
| ^(^[^34^](#_ENREF_34)^)^ | 1 | 1 | 1 | 0 | 1 | 1 | 1 | 1 | 1 | 1 | 9 |
| ^(^[^59^](#_ENREF_59)^)^ | 1 | 1 | 1 | 0 | 1 | 0 | 1 | 1 | 1 | 1 | 8 |
| ^(^[^57^](#_ENREF_57)^)^ | 1 | 1 | 1 | 0 | 1 | 0 | 1 | 1 | 1 | 0 | 7 |
| ^(^[^35^](#_ENREF_35)^)^ | 1 | 1 | 1 | 0 | 1 | 1 | 1 | 1 | 1 | 1 | 9 |
| ^(^[^62^](#_ENREF_62)^)^ | 1 | 1 | 1 | 0 | 1 | 1 | 1 | 1 | 1 | 0 | 8 |
| ^(^[^60^](#_ENREF_60)^)^ | 1 | 1 | 1 | 0 | 1 | 0 | 1 | 1 | 1 | 0 | 7 |
| ^(^[^50^](#_ENREF_50)^)^ | 1 | 1 | 1 | 0 | 1 | 1 | 1 | 1 | 1 | 0 | 8 |
| ^(^[^70^](#_ENREF_70)^)^ | 1 | 1 | 1 | 0 | 0.5 ^a^ | 0 | 1 | 1 | 1 | 0 | 6.5 |
| ^(^[^37^](#_ENREF_37)^)^ | 1 | 1 | 1 | 0 | 1 | 1 | 1 | 1 | 1 | 1 | 9 |
| ^(^[^45^](#_ENREF_45)^)^ | 1 | 1 | 1 | 0 | 1 | 0 | 1 | 1 | 1 | 1 | 8 |
| ^(^[^42^](#_ENREF_42)^)^ | 1 | 1 | 1 | 1 | 1 | 0 | 1 | 1 | 1 | 1 | 9 |
| ^(^[^54^](#_ENREF_54)^)^ | 1 | 1 | 1 | 0 | 1 | 1 | 1 | 1 | 1 | 1 | 9 |
| ^(^[^31^](#_ENREF_31)^)^ | 1 | 1 | 1 | 1 | 1 | 0 | 1 | 1 | 1 | 1 | 9 |
| ^(^[^56^](#_ENREF_56)^)^ | 1 | 1 | 1 | 0 | 1 | 0 | 1 | 1 | 1 | 0 | 7 |
| ^(^[^44^](#_ENREF_44)^)^ | 1 | 1 | 1 | 1 | 1 | 0 | 1 | 1 | 1 | 1 | 9 |
| ^(^[^41^](#_ENREF_41)^)^ | 1 | 1 | 1 | 1 | 1 | 0 | 1 | 1 | 1 | 1 | 9 |
| ^(^[^32^](#_ENREF_32)^)^ | 1 | 1 | 1 | 1 | 1 | 1 | 1 | 1 | 1 | 1 | 10 |
| ^(^[^46^](#_ENREF_46)^)^ | 1 | 1 | 1 | 0 | 1 | 1 | 1 | 1 | 1 | 1 | 9 |
| ^(^[^47^](#_ENREF_47)^)^ | 1 | 1 | 1 | 0 | 1 | 0 | 1 | 1 | 1 | 1 | 8 |
| ^(^[^52^](#_ENREF_52)^)^ | 1 | 1 | 1 | 0 | 1 | 0 | 1 | 1 | 1 | 1 | 8 |
| ^(^[^64^](#_ENREF_64)^)^ | 1 | 1 | 1 | 0 | 1 | 0 | 1 | 1 | 1 | 1 | 8 |
| ^(^[^48^](#_ENREF_48)^)^ | 1 | 1 | 1 | 0 | 1 | 0 | 1 | 1 | 1 | 1 | 8 |
| ^(^[^53^](#_ENREF_53)^)^ | 1 | 1 | 1 | 0 | 1 | 1 | 1 | 1 | 1 | 1 | 9 |

^a^ information on fasting condition not provided

^b^ information on anaesthesia not provided
