# Supplementary material for: Effects of diets containing proteins from fish muscles or fish by-products on the circulating cholesterol concentration in rodents: a systematic review and meta-analysis
Source: Br J Nutr. 2022 Oct 21;130(3):389–410. doi: 10.1017/S000711452200349X (PMC10331438; doi:10.1017/S000711452200349X)
Supplement: Supplementary file 1 [file S000711452200349Xsup.zip › S000711452200349Xsup004.docx]

**Supplementary Table 4:** Summary of subgroup meta-analyses for the effects of dietary intake of proteins from fish muscles or fish by-products on the circulating TC concentration

|  | Number of rodents included | Test for overall effect | Mean difference (95% CI), mmol/l | Tests for heterogeneity |
| --- | --- | --- | --- | --- |
| *The main meta-analysis:* | |  |  |  |
| All studies included (the main meta-analysis) | 935 | P < 0.00001  Z = 5.10 | -0.24 (-0.34, -0.15) | Chi^2^ = 222.05 P<0.00001  I^2^ = 71%, |
| *Subgroup analysis: muscles or by-products: P 0.37* | | | |  |
| Diets containing proteins from fish muscles | 680 | P < 0.00001  Z = 5.16 | -0.27 (-0.37, 0.17) | Chi^2^ = 149.51 P<0.00001  I^2^ = 72% |
| Diets containing proteins from fish by-products | 255 | P = 0.16  Z = 1.40 | -0.16 (-0.38, 0.06) | Chi^2^ = 72.47 P<0.00001  I^2^ = 71% |
| *Subgroup analysis: members of the Gadidae, the Salmonidae or the Clupeidae family: P 0.73* | | | | |
| Diets containing proteins from *Gadidae* family | 514 | P < 0.00001  Z = 5.88 | -0.28 (-0.38, -0.19) | Chi^2^ = 75.14  P<0.00001  I^2^ = 60% |
| Diets containing proteins from *Salmonidae* family | 205 | P = 0.05  Z = 1.94 | -0.24 (-0.48, 0.00) | Chi^2^ = 72.93  P<0.00001  I^2^ = 78% |
| Diets containing proteins from *Clupeidae* family | 150 | P = 0.44  Z = 0.77 | -0.14 (-0.50, 0.22) | Chi^2^ = 25.40  P=0.008  I^2^ = 57% |
| *Subgroup analysis: total or partial replacement of casein: P 0.29* | | | |  |
| Total replacement of casein with proteins from fish | 443 | P = 0.0002  Z = 3.73 | -0.32 (-0.49, -0.15) | Chi^2^ = 102.77  P < 0.00001  I^2^ = 74% |
| Replacement of ≤ 50% of casein with proteins from fish | 425 | P < 0.0001  Z = 4.34 | -0.24 (-0.35, -0.13) | Chi^2^ = 92.49  P < 0.00001  I^2^ = 66% |
| *Subgroup analysis: addition of cholesterol/cholate, or no cholesterol/cholate addition in diets fed to Zucker fa/fa rats or ob/ob mice: P 0.0002* | | | | |
| Diets enriched with cholesterol/cholate, or no cholesterol/cholate addition fed to *fa/fa* rats or *ob/ob* mice | 409 | P < 0.00001  Z = 5.65 | -0.53 (-0.72, -0.35) | Chi^2^ = 101.10  P < 0.00001  I^2^ = 75% |
| Diets not enriched with cholesterol/cholate | 526 | P = 0.02  Z = 2.36 | -0.13 (-0.23, -0.02) | Chi^2^ = 13.81  P < 0.00001  I^2^ = 71% |

The intervention and comparator groups were compared using the random effects inverse-variance model using Review Manager v. 5.4.1
